# Ethylene and hydrogen peroxide regulate formation of a sterol-enriched domain essential for wall labyrinth assembly in transfer cells

**DOI:** 10.1093/jxb/erz003

**Published:** 2019-01-14

**Authors:** Hui-Ming Zhang, Luke B Devine, Xue Xia, Christina E Offler, John W Patrick

**Affiliations:** School of Environmental and Life Sciences, University of Newcastle, Newcastle NSW, Australia

**Keywords:** Ethylene, hydrogen peroxide, Ca^2+^ signal, plasma membrane, sterol-enriched domain, *trans*-differentiation, transfer cell, wall labyrinth

## Abstract

Transfer cells (TCs) facilitate high rates of nutrient transport into, and within, the plant body. Their transport function is conferred by polarized wall ingrowth papillae, deposited upon a specialized uniform wall layer, that form a scaffold supporting an amplified area of plasma membrane enriched in nutrient transporters. We explored the question of whether lipid-enriched domains of the TC plasma membrane could serve as organizational platforms for proteins regulating the construction of the intricate TC wall labyrinth using developing *Vicia faba* cotyledons. When these cotyledons are placed in culture, their adaxial epidermal cells *trans*-differentiate to a TC phenotype regulated by auxin, ethylene, extracellular hydrogen peroxide (_apo_H_2_O_2_), and cytosolic Ca^2+^ ([Ca^2+^]_cyt_) arranged in series. Staining cultured cotyledons with the sterol-specific dye, Filipin III, detected a polarized sterol-enriched domain in the plasma membrane of their *trans-*differentiating epidermal transfer cells (ETCs). Ethylene activated sterol biosynthesis while extracellular _apo_H_2_O_2_ directed sterol-enriched vesicles to fuse with the outer periclinal region of the ETC plasma membrane. The sterol-enriched domain was essential for generating the [Ca^2+^]_cyt_ signal and orchestrating construction of both the uniform wall layer and wall ingrowth papillae. A model is presented outlining how the sterol-enriched plasma membrane domain forms and functions to regulate wall labyrinth assembly.

## Teaser

Ethylene and extracellular hydrogen peroxide co-regulate formation of a sterol-enriched plasma membrane domain in *trans*-differentiating epidermal transfer cells that functions to co-ordinate construction of their wall labyrinth.

## Introduction

Transfer cells (TCs) exhibit an enhanced capacity for nutrient transport conferred by an amplified plasma membrane (PM) surface area, enriched in membrane nutrient transporters, supported on an intricate wall labyrinth (Offler *et al.*, 2003). Consistent with this functional capacity, TCs are located at bottlenecks for nutrient transport between apo-symplasmic compartments. For example, TCs can occur at interfaces between soil/root, host/biotroph, and maternal/filial tissues of developing seeds or at sites of nutrient loading/unloading of vascular pipelines (Offler *et al.*, 2003; Andriunas *et al.*, 2013). The tight functional relationship between the presence of a wall labyrinth (i.e. PM amplification) and enhanced nutrient transport capacity is graphically illustrated by compromised filling of eudicot and monocot seed mutants in which wall labyrinth formation of their TCs is attenuated. That the shrivelled seed phenotype of these TC mutants are displayed in a number of major crop species, including cereals and grain legumes, underscores the important role TCs play in determining crop yields (Andriunas *et al.*, 2013).

Wall labyrinths of TCs are one of two principal architectural types—reticulate or flange, or, in a few instances, both wall architectures occur in the same cell with the reticulate type deposited on flanges (Andriunas *et al.*, 2013). The reticulate architecture provides the greatest plasticity for amplifying PM surface areas. The degree of reticulation ranges from a single layer of discrete or branched wall ingrowth (WI) papillae to repeating fenestrated layers of WI papillae that branch and fuse (Andriunas *et al.*, 2013). The selective advantage of the reticulate wall labyrinth design is illustrated by its presence in species of all plant taxa from algae to angiosperms (Offler *et al.*, 2003). As a consequence, most studies have focused on reticulate TCs with their wall labyrinths comprised of a uniform wall layer (UWL) from which WI papillae arise (Xia *et al.*, 2017).

To understand the mechanisms and regulatory signals responsible for constructing reticulate wall labyrinths, our investigations have focused on the assembly of the UWL and the first round of construction of WI papillae using cultured cotyledons of *Vicia faba*. On being transferred to culture, the adaxial epidermal cells of the *V. faba* cotyledons undergo rapid (hours) *trans*-differentiation to a TC morphology and function comparable with their *in planta* abaxial epidermal TC (ETC) counterparts (Andriunas *et al.*, 2013). In both adaxial and abaxial ETCs, wall labyrinths are polarized to their outer periclinal walls. An inductive signalling cascade, driving the *trans*-differentiation process, is initiated by an auxin spike in the adaxial epidermal cells (Dibley *et al.*, 2009), triggering ethylene production (Zhou *et al.*, 2010). The ethylene signal elicits a polarized burst in extracellular hydrogen peroxide (_apo_H_2_O_2_) production that switches on, and provides the positional cue for, the localized deposition of the UWL to the outer periclinal wall (Andriunas *et al.*, 2012; Xia *et al.*, 2012). Acting co-operatively, ethylene and _apo_H_2_O_2_ induce the formation of a polarized cytosolic Ca^2+^ ([Ca^2+^]_cyt_) signal (Zhang *et al.*, 2015*b*) shaped into plumes of elevated [Ca^2+^]_cyt_ by enhanced fluxes of Ca^2+^ through PM-localized clusters of Ca^2+^-permeable channels surrounded by Ca^2+^-ATPases (Zhang *et al.*, 2015*a*). The inward-directed [Ca^2+^]_cyt_ plumes cause re-modelling of the actin cytoskeleton into a spatial configuration that trafficks vesicle cargoes, containing wall building materials, to defined loci on the outer surface of the UWL for assembly of WI papillae (Zhang *et al.*, 2017*a*).

As described above, assembly of reticulate wall labyrinths is dependent upon mechanism(s) that localize _apo_H_2_O_2_ and [Ca^2+^]_cyt_ signals, together with cell wall deposition, to the outer periclinal wall of the *trans*-differentiating ETCs. It is well established that sterol- and sphingolipid-enriched PM domains selectively compartment proteins participating in signalling pathways and cell wall biosynthesis (e.g. Schrick *et al.*, 2012; Tapken and Murphy, 2015; Iswanto and Kim, 2017; Han *et al.*, 2018). In this context, transcriptomic analyses have found that genes encoding sterol and sphingolipid biosynthetic enzymes, along with a microdomain marker protein, remorin, were up-regulated during *trans*-differentiation of both flange and reticulate wall labyrinths (Thiel 2012; Zhang *et al.*, 2015*c*). However, until now, there is no direct evidence for the presence of lipid-enriched PM domains organizing signalling pathways or cell wall biosynthesis underpinning assembly of TC wall labyrinths. Nevertheless, precedents for such domains are evident in analogous systems. For instance, sterol-enriched macrodomains are found in the PM lining tips of developing pollen tubes and root hairs (Liu *et al.*, 2009; Ovecka *et al.*, 2010). Significantly, sterol sequestration to the cytosol disrupted polarization of these macrodomains, and depressed activities of tip-located respiratory burst oxidase homologues (RBOHs) producing a reactive oxygen species (ROS) signal that in turn dissipated the apically orientated [Ca^2+^]_cyt_ gradient. The consequence was arrested tip growth of elongating pollen tubes (Liu *et al.*, 2009). Similarly, precedents for localizing callose synthases to deposit callose sheaths of WI papillae (Vaughn *et al.*, 2007) are PM domains that regulate callose deposition to build defence papillae and control gating of plasmodesmal pores (Faulkner *et al.*, 2015; Iswanto and Kim, 2017).

Given these precedents, we explored the proposition that lipid-enriched domains in the PM of ETCs of *V. faba* cotyledons contribute to assembling their reticulate wall labyrinths. To this end, we show that the PM lining the outer periclinal region of developing ETCs was selectively and rapidly enriched in sterols during the early phases of *trans*-differentiation to a TC morphology. Ethylene switched on sterol biosynthesis and _apo_H_2_O_2_ directed trafficking of sterol-enriched membrane vesicles to fuse with the PM lining the outer periclinal ETC region. In turn, the sterol-enriched domain (SED) within the PM directly regulated assembly of the UWL. Influence over formation of WI papillae was mediated through the SED functioning as an organizing platform for Ca^2+^-permeable channels to generate the polarized plumes of elevated [Ca^2+^]_cyt_ that direct construction of WI papillae.

## Materials and methods

### Plant growth conditions

*Vicia faba* L. cv. Fiord plants were raised under controlled environmental conditions according to Zhou *et al.* (2010).

### Cotyledon culture

*Vicia faba* cotyledons were aseptically cultured on a modified Murashige and Skoog (MS) liquid medium (Murashige and Skoog, 1962; see Andriunas *et al.*, 2012). Sister cotyledons were divided between MS medium with or without pharmacological agents and held in the dark at 4 °C for 4 h to allow their penetration before being cultured at 26 °C for a further 15 h unless stated otherwise. Each pharmacological agent was applied at a concentration that did not negatively impact cell viability as verified by staining cotyledon sections with 0.1% (w/v) tetrazolium blue.

### Visualization of sterol-enriched membrane domains

Sterol-enriched membrane domains in ETCs were visualized by staining freshly prepared tissue sections of cotyledons with the UV fluorescent probe, Filipin III (hereafter referred to as Filipin), that binds to the 3'-β-OH group of sterols, following a protocol for live cell imaging described by Boutté *et al.* (2011) modified as follows. Cotyledons, sampled directly from plants or following culture with or without pharmacological treatments, were hand sectioned along their transverse axis to provide ready access of all cells to the Filipin stain (Boutté *et al.*, 2011). Tissue sections (~100 μm in thickness) were immediately incubated in 30 µM Filipin dissolved in MES-buffered MS medium (pH 5.8) containing 10 mM sucrose for 30 min at 4 °C in the dark to prevent wound responses, Filipin photobleaching, and sterol-mediated endocytosis. Holding the sectioned tissues at 4 °C was employed to minimize confounding effects on sterol abundance and distribution in the ETCs that may have been introduced by sectioning-induced wound responses and by Filipin impacting endocytosis and sterol sequestration, while darkness reduced photobleaching of Filipin fluorescence (Boutté *et al.*, 2011). The stained sections were washed in the MS/sucrose solution for 3 min at 4 °C, changing the wash solution at 1 min intervals, to remove unbound Filipin. Thereafter, the stained sections were mounted in pre-chilled MS/sucrose solution and observed with an Olympus FluoView FV1000 confocal laser-scanning microscope (CLSM) using a 60× oil-immersion objective (NA 1.25). Filipin was excited with a 405 nm UV laser source (50 mW, laser power set to 15%) and emitted fluorescence captured at 425–485 nm using a photomultiplier gain of 700 V. Total fluorescence of Filipin, measured as pixels in specified regions of the PM of ETCs and storage parenchyma cells (SPCs), was estimated using the freehand selection tool in ImageJ (http://rsbweb.nih.gov/ij/), and corrected for background fluorescence measured in unstained cells.

To determine the subcellular localization of Filipin fluorescence, whole cotyledons were stained with 20 µM RH-414, a PM marker (Hafke *et al.*, 2007), for 30 min prior to hand sectioning and Filipin staining. To visualize RH-414-stained PMs, the co-stained tissue sections were exposed to an excitation wavelength of 559 nm (15 mW, laser power set to 25%) and emitted fluorescence captured at 625–725 nm using a photomultiplier gain of 500 V.

### Transmission and scanning electron microscopy

Ultrathin transverse sections of ETCs were visualized with a JEOL 1200 EX II transmission electron microscope (JOEL, Japan) as previously described (Zhang *et al.*, 2015*d*). Average UWL thicknesses were estimated from measures of UWL cross-sectional areas divided by their corresponding widths (i.e. nm^2^ nm^−1^=nm) using ImageJ software (http://rsbweb.nih.gov/ij/). A Zeiss VP field emission scanning electron microscope (FESEM; Zeiss, Germany) was used to visualize WI papillae on the cytoplasmic faces of the outer periclinal walls of fractured ETCs, prepared as described by Zhang *et al.* (2015*d*).

### Monitoring vesicle trafficking

To assess the impact of SEDs on vesicle trafficking during different phases of wall labyrinth assembly, transverse free-hand sections of cultured cotyledons were stained with FM4-64FX (Molecular Probes, Eugene, OR, USA; see Zhang *et al*, 2017*a*). Total fluorescence of specified ETC regions was measured using ImageJ software and corrected against PM fluorescence, as described in Zhang *et al.* (2017*a*).

### Measurement of _apo_H_2_O_2_ flux and distribution

Flux (pmol H_2_O_2_ min^−1^ mm^−2^ of the adaxial cotyledon surface) of _apo_H_2_O_2_ generated in the outer periclinal wall of ETCs was measured using Amplex Red reagent (10-acetyl-3. 7-dihydrophenoxazine; Invitrogen, Australia) as described by Andriunas *et al.* (2012).

The histochemical reagent, 3,3'-diaminobenzidine (DAB; Sigma, Australia), was used to localize _apo_H_2_O_2_ distribution based on its ability to generate a stable, insoluble brown-coloured precipitate upon binding with H_2_O_2_, as described by Andriunas *et al.* (2012). Fresh cotyledon sections (100 μm in thickness) were viewed under bright field with a Zeiss Axiophot microscope and ETC images were recorded with a Zeiss AxioCam HRc camera using Axiovision software. Images were processed through Photoshop CS6 level command with input levels adjusted to 156–237 in both the negative control and DAB-stained sections to an identical setting so that the image of the brown DAB stain was intensified. Absolute pixel numbers of the DAB stain in each cell wall region, corrected for background, were determined using Image J in RawIntDen under Integrated Density measure (https://imagej.nih.gov/ij/docs/menus/analyze.html).

### Visualization of cytosolic calcium and fluorescently labelled Ca^2+^-permeable channels

Estimates of [Ca^2+^]_cyt_ were obtained by loading cultured cotyledons with a membrane-permeable Ca^2+^-sensitive dye, Oregon Green BAPTA-1 acetoxymethyl ester (OGB-1), while the cellular distribution of Ca^2+^-permeable channels relied on staining cultured cotyledons with DM-BODIPY(–)-dihydropyridine (fl-DHP; Invitrogen, USA; see Zhang *et al.*, 2015*a*).

Thereafter transverse hand-cut sections were stained with tetrazolium blue to identify viable cells for microscope observations and counterstained with 0.1% (w/v) Calcofluor White to outline the ETC walls. An Olympus FV1000 CLSM set with a 405 nm laser and a 440–490 nm emission filter set was used to visualize Calcofluor white fluorescence, while a 473 nm laser and a 510–550 nm emission filter set was used to visualize OGB-1 and fl-DHP fluorescence. Fluorescence densities (pixels per unit area) of OGB-1 in the outer periclinal cytosol of ETCs were measured using ImageJ and converted to estimates of [Ca^2+^]_cyt_ using the calibration curve presented in Zhang *et al.* (2015*a*). The utility of the pre-existing calibration curve was verified by finding that it yielded identical [Ca^2+^]_cyt_ estimates to those reported by Zhang *et al.* (2015a, b). Total fluorescence of fl-DHP in specified regions in the ETCs was measured using ImageJ.

### RNAseq expression analysis

A transcriptomic database for *trans*-differentiating ETCs and their underlying SPCs of cultured *V. faba* cotyledons, annotated in Mapman Mercator and the Kyoto Encyclopedia of Genes and Genomes (KEGG) (Zhang *et al.*, 2017*b*), was used to investigate expression profiles of transcripts encoding proteins participating in phytosterol and sphingolipid biosynthesis and metabolism (Michaelson *et al.*, 2016; Sonawane *et al.*, 2016; Valitova *et al.*, 2016), the cytosolic ethylene insensitive2 (EIN2) pathway (Salehin and Estelle, 2015; Zheng and Zhu, 2016), and vesicle trafficking (Kim and Brandizzi, 2012; Paez Valencia *et al.*, 2016). Identified transcripts with RPKMs (reads per kilobase of transcript per million mapped reads) >1 at 3 h or 12 h of cotyledon culture were selected for subsequent analysis. Functions of the encoded proteins were inferred by best-fit percentage amino acid alignment with their closest Arabidopsis homologue using TAIR10 and Araport 11 databases. The impact of ethylene and _apo_H_2_O_2_ on expression profiles of these transcripts was assessed as described in Zhang *et al.* (2017*b*).

## Results

### The outer periclinal region of the plasma membrane of *trans*-differentiating epidermal transfer cells is sterol enriched

**Fig. 1.**
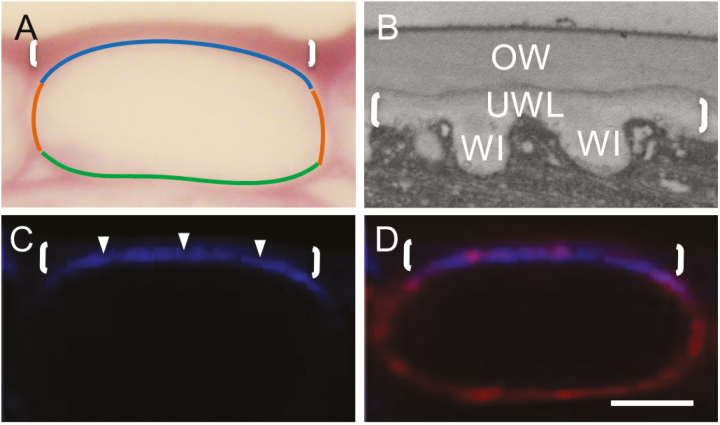
Micrographs illustrating the morphological characteristics of *trans*-differentiating epidermal transfer cells (ETCs) of cultured *V. faba* cotyledons and co-localization of Filipin staining with their plasma membrane. (A and B) Light (A) and transmission electron (B) micrographs of transverse sections of 15 h cultured ETCs. Note that the wall labyrinth is polarized to the outer periclinal region (A), is deposited on the original primary wall (OW) in (B), and is composed of a uniform wall layer (UWL, bracketed) from which wall ingrowth (WI) papillae arise. Identified ETC regions referred to in this study are marked on (A), namely outer periclinal (blue line), anticlinal (rusty brown line), and inner periclinal (green line). (C and D) CLSM images of transverse sections of ETCs stained with Filipin to detect sterol-enriched domains (fluorescence indicated by arrowheads in C), co-stained with the plasma membrane tracker RH-414, and presented as an overlay (D) with brackets delineating the outer periclinal wall. Scale bar=10 µm in (A), 500 nm in (B), and 5 µm in (C) and (D).

By 15 h of cotyledon culture, the *trans*-differentiating ETCs had deposited a wall labyrinth across the outer periclinal region of their original primary wall that is thicker than the anticlinal and inner periclinal walls (Fig. 1A). The wall labyrinth is comprised of a UWL from which WI papillae arise (Fig. 1B). Filipin-stained ETCs of the 15 h cultured cotyledons exhibited a strong fluorescent band located in their outer periclinal region (Fig. 1C). Co-localization of fluorescence emanating from bound Filipin with the PM tracker, RH-414, in both turgid and plasmolysed ETCs pointed to Filipin binding to the PM of *trans*-differentiating ETCs (Fig. 1D; Supplementary Fig. S1 at *JXB* online). Consistent with this conclusion was finding that the intracellular distribution of Filipin fluorescence levels was unaffected by treating cotyledons with the vesicle trafficking inhibitor, brefeldin A (BFA; Supplementary Table S1). This result excludes localization of bound Filipin to vesicles that would be distributed evenly throughout the ETC cytosol by cytoplasmic streaming.

**Fig. 2.**
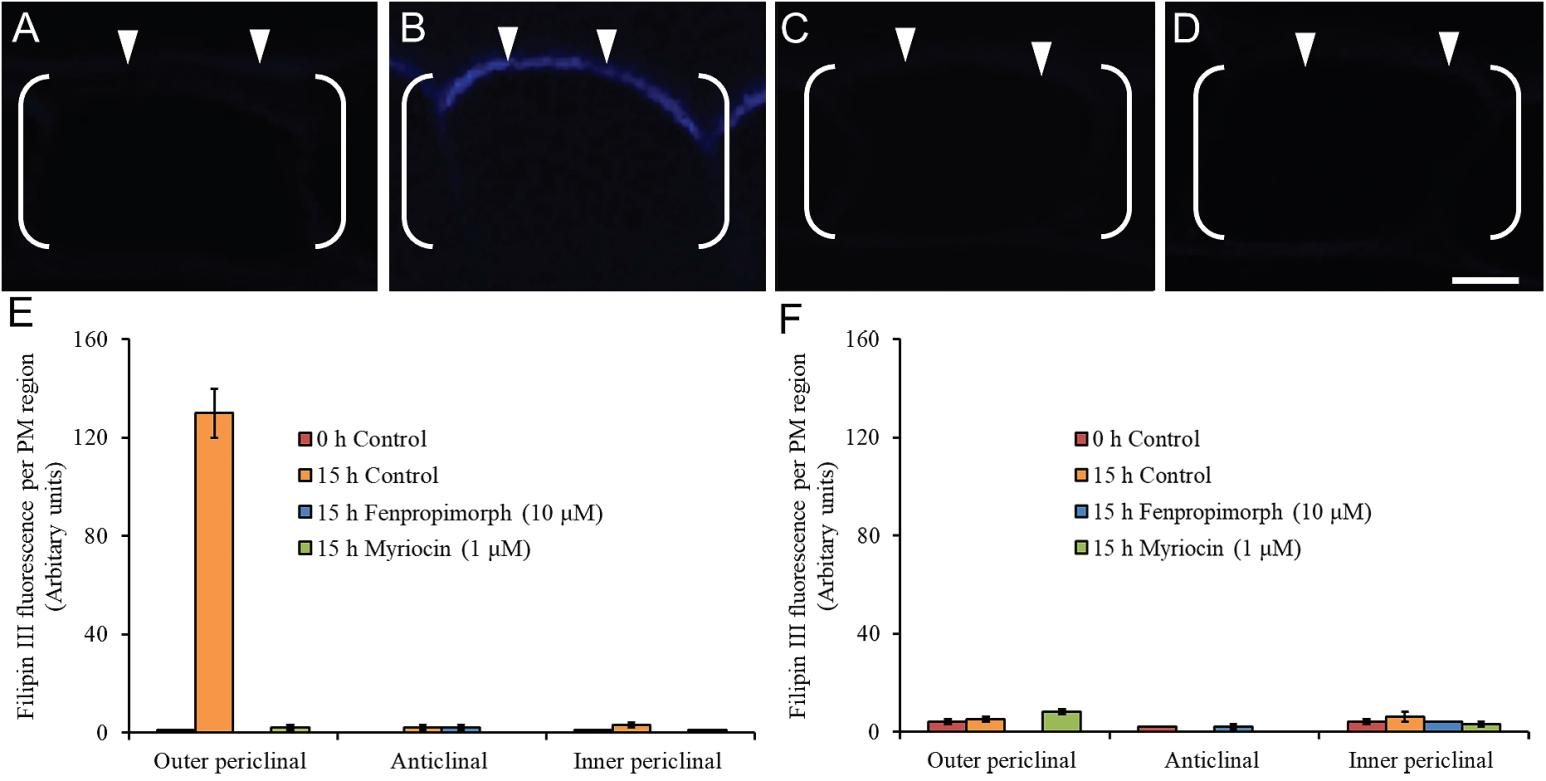
Impact of inhibitors of sterol and sphingolipid biosynthesis on the intracellular distribution of Filipin fluorescence in *trans*-differentiating epidermal transfer cells (ETCs) and storage parenchyma cells (SPCs) of cultured *V. faba* cotyledons. (A–D) CLSM images of transverse sections of ETCs prepared from cotyledons that were (A) freshly harvested or (B–D) cultured on MS medium for 15 h in the (B) absence or (C) presence of 10 µM fenpropimorph or (D) 1 µM myriocin. The ETCs are bracketed, and arrowheads indicate the position of their outer periclinal region. Scale bar=5 µm. (E, F) Filipin fluorescence measured as total pixels detected in outer periclinal, anticlinal, and inner periclinal PM regions of (E) ETCs and (F) SPCs treated with ±10 µM fenpropimorph or ±1 µM myriocin. Data are means ±SEs from four replicate cotyledons; 20 cells per cotyledon.

The 43-fold enhanced fluorescence levels of bound Filipin located in the ETC outer periclinal region of their PM (Fig. 2A versus Fig. 2B, E) was absent from the underlying SPCs in which very low Filipin fluorescence levels were spread evenly across their entire PM (Fig. 2F). The slightly higher fluorescence values recorded in the anticlinal and inner periclinal PM regions of SPCs compared with those of the ETCs was proportionate to their larger size (Fig. 2E versus Fig. 2F; for more details, see Supplementary Table S2).

Blocking formation of the polarized band of Filipin fluorescence by culturing cotyledons on media containing biosynthesis inhibitors of sterol (fenpropimorph, Yang *et al.*, 2013) or sphingolipids (myriocin, Michaelson *et al.*, 2016) indicated that the fluorescence emanated from Filipin bound to an SED located in the outer periclinal region of the ETC PM (Fig. 2B versus Fig. 2C–E); a conclusion confirmed using the PM sterol stripping drug, methyl-β-cyclodextrin, and the sphingolipid biosynthesis inhibitor, fumonsin B1 (Supplementary Table S3; Yang *et al.*, 2013). The possibility that Filipin stained brassinosteroids was eliminated as inhibiting their biosynthesis with triadimefon or propiconazole (Yang *et al.*, 2013) exerted no impact on Filipin fluorescence (Supplementary Table S3).

Temporal accumulation of the polarized SED in the PM of ETCs (Fig. 2E) was monitored at specified times across 15 h of cotyledon culture by recording percentages of cells exhibiting the band of intense Filipin fluorescence and mean fluorescence levels per ETC (Supplementary Fig. S2A and B, respectively). Both parameters increased from the onset of cotyledon culture to asymptote at 6 h (Supplementary Fig. S2).

### Ethylene regulates biosynthesis, while _apo_H_2_O_2_ determines the intracellular distribution, of the sterol-enriched domain

To test if any of the known signals (auxin, ethylene, _apo_H_2_O_2_, or calcium) regulating wall labyrinth assembly was responsible for switching on sterol biosynthesis or determining SED polarization (Fig. 2), cotyledons were cultured on media containing blockers of auxin [*p*-chlorophenoxyisobutyric acid (PCIB), Dibley *et al.*, 2009], ethylene [aminoethoxyvinylglycine (AVG), Zhou *et al.*, 2010], _apo_H_2_O_2_ [ascorbic acid (AA), Andriunas *et al.*, 2012], and [Ca^2+^]_cyt_ (nifedipine, Zhang *et al.*, 2015*a*). Filipin fluorescence levels were measured in outer periclinal, anticlinal, and inner periclinal PM regions to assess impacts on SED polarization. The summed fluorescence levels in these regions provided estimates of sterol biosynthesis per ETC.

Only AVG caused a 96% depression in total Filipin fluorescence per ETC (Table 1), indicating that ethylene up-regulated sterol biosynthesis, a conclusion consistent with elevated ETC sterol levels in response to a 54% enhanced ethylene production in the presence of the ethylene precursor, 1-aminocyclopropane-1-carboxylic acid (ACC) (Table 1; Zhou *et al.*, 2010). Our data do not provide any insight as to why the increase in sterol levels was confined to the anticlinal and inner periclinal PM domains. In comparison, while sterol biosynthesis per ETC was not attenuated, the Filipin fluorescence band was depolarized in ETCs of cotyledons cultured on AA, a H_2_O_2_ scavenger, known to quench the polarized _apo_H_2_O_2_ signal (Andriunas *et al.*, 2012). However, the ETC level, and polarity, of the Filipin fluorescence was retained when the spike in [Ca^2+^]_cyt_ was blocked on cotyledon exposure to the Ca^2+^ channel inhibitor, nifedipine (Table 1; Zhang *et al.*, 2015*a*).

**Table 1.** Effect of auxin, ethylene, _apo_H_2_O_2_, and Ca^2+^ signalling on the plasma membrane (PM) sterol-enriched domain formation and distribution in *trans*-differentiating epidermal transfer cells (ETCs) of cultured *V. faba* cotyledons

Cotyledon treatment	Active signal(s)	Inhibited signal(s)	Filipin fluorescence (arbitrary units) in ETC:			
			Outer periclinal PM	Anticlinal PM	Inner periclinal PM	Total
Control	IAA, C_2_H_4_, _apo_H_2_O_2_, Ca^2+^	None	131±5	2±1	0±0	132±5
PCIB (100 μM)+ACC (100 μM)	C_2_H_4_, _apo_H_2_O_2_, Ca^2+^	IAA	136±6	39±3	33±2	208±8
AVG (100 μM)+H_2_O_2_ (10 μM)	IAA, _apo_H_2_O_2_, Ca^2+^	C_2_H_4_	0±0	4±1	1±0	5±1
AVG (100 μM)+ACC (100 μM)	IAA, C_2_H_4_, _apo_H_2_O_2_, Ca^2+^	None	142±7	38±2	28±3	208±10
Ascorbic acid (10 mM)	IAA, C_2_H_4_	H_2_O_2_, Ca^2+^	48±4	43±3	49±4	140±9
Nifedipine (100 μM)	IAA, C_2_H_4_, _apo_H_2_O_2_	Ca^2+^	128±9	5±1	0±0	133±10

Cotyledons were cultured for 15 h on MS medium in the absence or presence of various combinations of the auxin action inhibitor, *p*-chlorophenoxyisobutyric acid (PCIB), ethylene biosynthesis inhibitor, aminoethoxyvinylglycine (AVG), ethylene precursor, 1-aminocyclopropane-1-carboxylic acid (ACC), _apo_H_2_O_2_ scavenger, ascorbic acid, and the DHP-receptor Ca^2+^ channel blocker, nifedipine. Thereafter, transverse sections of treated cotyledons were stained with Filipin. Fluorescence was measured as total pixels in specified regions of the ETC PM. Data are means ±SEs from four replicate cotyledons; 20 cells per cotyledon.

### Ethylene regulates biosynthesis of the sterol-enriched domain

To better understand how ethylene regulated ETC sterol biosynthesis, an experiment was designed to distinguish ethylene action at the various levels of molecular control. Here cotyledons were cultured under conditions with or without ethylene in the absence/presence of the RNA inhibitor, 6-methylpurine, or the protein inhibitor, cycloheximide, and treatment effects on Filipin fluorescence were determined (Supplementary Table S4). These data were then used to distil out actions of ethylene at the transcriptional, translational, and post-translational levels (for details, see Table 2 footnotes). The analysis demonstrated that ethylene acted primarily on translation of mRNA encoding sterol biosynthetic enzymes, with decreasing impacts at the transcriptional and post-translational levels (Table 2).

**Table 2.** Regulatory role of ethylene on sterol biosynthesis at the transcriptional, translational, and post-translational levels in *trans*-differentiating epidermal transfer cells (ETCs) of cultured *V. faba* cotyledons

Effect of ethylene on:	Filipin fluorescence/ETC (arbitrary units)
Transcription	33±13 (16±6%)
Translation	159±10 (77±5%)
Post-translation	14±4 (7±2%)

Data are derived from Supplementary Table S4 as follows. Fluorescence levels in the presence of ethylene were corrected by subtracting fluorescence in its absence. Effects of ethylene at the transcriptional, translational, and post-translational levels were estimated, respectively, from differences in total Filipin fluorescence levels detected in ETCs between cotyledons cultured on: (i) MS medium alone versus 6-methylpurine; (ii) 6-methylpurine versus cycloheximide; and (iii) cyclohexamide alone. The percentage contribution to sterol biosynthesis by each level of biological control is presented in parentheses.

Our RNA sequencing (RNAseq) data set was interrogated for ETC-specific differentially expressed genes (DEGs) encoding enzymes known to participate in the biosynthetic pathways for phytosterols (Sonawane *et al.*, 2016; Valitova *et al.*, 2016) and sphingolipids (Michaelson *et al.*, 2016). This analysis yielded two phytosterol and seven sphingolipid biosynthetic ETC-specific DEGs (Supplementary Table S5A). Ethylene had no impact on expression of the phytosterol biosynthetic DEGs, *Vf3βHSD* and *VfHMGS1* (Sonawane *et al.*, 2016). In the case of the seven sphingolipid biosynthetic ETC-specific DEGs, ethylene up-regulated expression of *Vfinositol phospoceramide synthase1* (*VfIPCS1*) operating in the pathway generating the largest group of plant sphingolipids, glycosyl inositol phosphoceramides (Michaelson *et al.*, 2016). The remaining sphingolipid DEGs were unresponsive to ethylene. Four DEGs encoded VfCB2SPT1, VfSPT2, VfTSC10B, and VfNCER, all of which could regulate sphingolipid levels. Amongst them, VfCB2SPT catalyses the first step in the sphingolipid biosynthetic pathway, VfTSC10B reduces 3-ketodihydrosphinganine into dihydrosphinganine, while VfNCER maintains homeostasis of the ceramide pool. The other two DEGs encoded VfSBH and VfÄ^8^-DS. These enzymes modify the long chain bases of sphingolipids by hydroxylation and desaturation, respectively, that in turn can alter their biological properties (Michaelson *et al.*, 2016).

EIN2 was recently shown also to act in the cytosol to mediate ethylene control of translation (Salehin and Estelle, 2015; Zheng and Zhu, 2016). Of downstream proteins associated with this pathway, expression of genes encoding ethylene-binding F-box proteins, *VfEBF1* and *VfEBF2*, were found to be ethylene sensitive between 3 h and 12 h of cotyledon culture (Supplementary Table S5B).

In the absence of ethylene, the SED was disassembled to background levels (Table 1). This response was associated with AVG increasing expression of two ETC-specific down-regulated DEGs, *VfPIP5K6* and *VfEpsin1* (Supplementary Table S5C). These respectively encode proteins that participate in clathrin-mediated endocytosis (Zhao *et al.*, 2010) and the late endosome/vacuolar lysis pathway (Song *et al.*, 2006).

### Extracellular hydrogen peroxide regulates intracellular distribution of the sterol-enriched domain

**Fig. 3.**
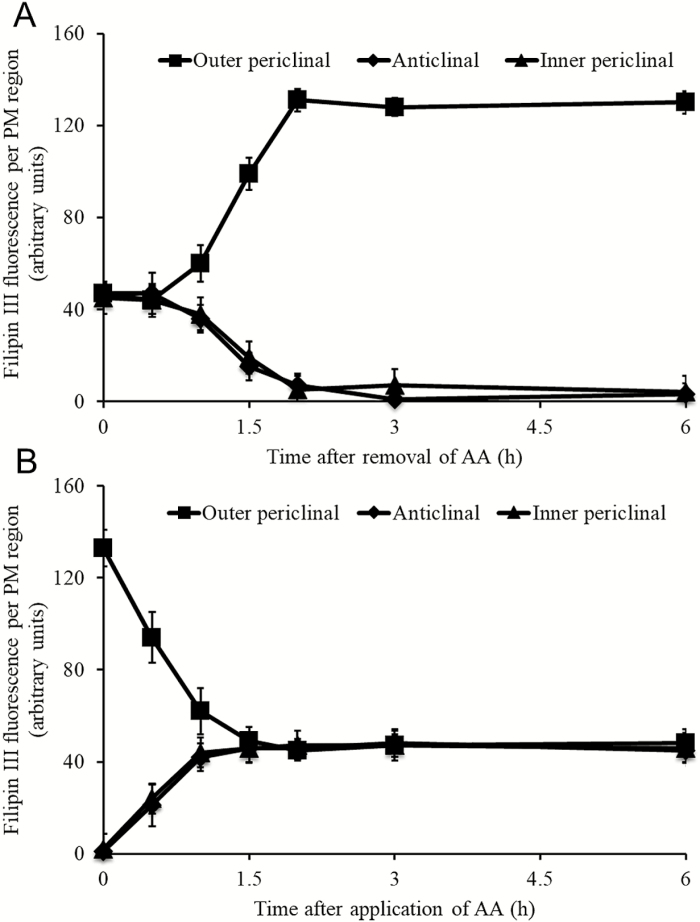
Temporal regulation of the plasma membrane (PM) sterol-enriched domain (SED) assembly and disassembly by the _apo_H_2_O_2_ signal in *trans*-differentiating epidermal transfer cells (ETCs) of cultured *V. faba* cotyledons. (A) To examine SED assembly, cotyledons were cultured on MS medium containing AA for 9 h at 26 °C, then washed 3×5 min in dH_2_O before being transferred to MS medium alone, for the specified times at 26 °C. (B) To examine SED disassembly, cotyledons were cultured on MS medium alone for 9 h and then transferred to MS medium containing the _apo_H_2_O_2_ scavenger, ascorbic acid (AA), for specified times at 26 °C. Thereafter, transverse sections of treated cotyledons were stained with Filipin. Fluorescence was measured as total pixels in specified regions of the ETC PM. Data at each culture time are means ±SEs from four replicate cotyledons; 20 cells per cotyledon.

The time course kinetics of _apo_H_2_O_2_-regulated assembly and disassembly of the SED were examined once Filipin fluorescence had acquired steady-state levels in ETCs at 9 h of cotyledon culture (Supplementary Fig. S2). For SED assembly, cotyledons were cultured on MS medium containing the _apo_H_2_O_2_ scavenger, AA, for 9 h. This treatment resulted in a depolarized intercellular distribution of Filipin fluorescence (Fig. 3A). Thereafter, on transfer to an AA-free MS medium, the polarity of Filipin fluorescence distribution was restored within 2 h (Fig. 3A). The polarity of the SED achieved by 9 h of cotyledon culture was depolarized completely within 1.5 h of transferring cotyledons to a MS medium containing AA (Fig. 3B). Together, these findings demonstrate that the _apo_H_2_O_2_ signal not only acts to polarize SED assembly, but is also essential in maintaining their polarized state by blocking disassembly.

The reliance of an _apo_H_2_O_2_-dependent polarized assembly/disassembly of the SED on the ETC cytoskeleton was tested by culturing cotyledons in the absence/presence of the microtubule- and actin-depolymerizing drugs, oryzalin (Zhang *et al.*, 2015*d*) and latrunculin B, respectively (Zhang *et al.*, 2017*a*). The experimental design was identical to that described for Fig. 3. The presence/absence of an intact cytoskeleton did not exert any influence over either _apo_H_2_O_2_-dependent assembly or disassembly of the polarized SED (Table 3).

**Table 3.** The dependence of _apo_H_2_O_2_ regulation of the plasma membrane (PM) sterol-enriched domain distribution on cortical microtubule (CMT) and actin cytoskeletons in *trans*-differentiating epidermal transfer cells (ETCs) of cultured *V. faba* cotyledons

Cotyledon treatment			Filipin fluorescence (arbitrary units) in ETC:			
			Outer periclinal PM	Anticlinal PM	Inner periclinal PM	Total
(A)	9 h AA (10 mM)+	6 h MS	138±8	0±0	3±1	141±8
		6 h LatB (100 nM)	134±7	4±1	1±0	139±8
		6 h Oryzalin (20 µM)	143±6	0±0	0±0	143±6
(B)	9 h MS+	6 h AA (10 mM)	42±3	42±2	43±3	127±6
		6 h AA (10 mM) +LatB (100 nM)	46±5	42±4	44±5	132±8
		6 h AA (10 mM) +Oryzalin (20 µM)	40±5	45±5	41±3	126±8

(A) Cotyledons were cultured on MS medium containing the _apo_H_2_O_2_ scavenger, ascorbic acid (AA), for 9 h at 26 °C, then washed 3×5 min in dH_2_O before being transferred to MS medium in the absence/presence of the CMT-depolymerizing drug, oryzalin, or the actin-depolymerizing drug, latrunculinB (LatB), for 4 h at 4 °C followed by a further 6 h of culture at 26 °C.

(B) Cotyledons were cultured on MS medium alone for 9 h before being transferred to MS medium containing AA in the absence/presence of oryzalin or LatB for 4 h at 4 °C for a further 6 h of culture at 26 °C.

Thereafter, transverse sections of treated cotyledons were stained with Filipin. Fluorescence was measured as total pixels in specified regions of the ETC PM. Data are means ±SEs from four replicate cotyledons; 20 cells per cotyledon.

Whether _apo_H_2_O_2_-regulated assembly and disassembly of the SED depended upon vesicle trafficking and endocytosis of sterol-enriched membrane vesicles was explored using the experimental plan described for Fig. 3 augmented by exposing cotyledons during 9–15 h of culture to media also containing dynasore (endocytosis inhibitor; Zhang *et al.*, 2017*a*). The resulting distribution of Filipin fluorescence is consistent with endocytosis blocking both _apo_H_2_O_2_-driven SED assembly (Supplementary Table S6A) and SED disassembly in the absence of _apo_H_2_O_2_ (Supplementary Table S6B).

The above findings point to _apo_H_2_O_2_ activating vesicle docking and inactivating endocytosis. Our RNAseq data set (see Supplementary Table S7) identified _apo_H_2_O_2_-sensitive expression of genes encoding proteins that function in the endomembrane secretory pathway from vesicle biogenesis in the Golgi/*trans-*Golgi network (*VfSYP32*, *VfSEC22*, *Vfβ-COP*, *Vfγ-COP*, and *VfAGD5*; Kim and Brandizzi, 2012; Arakel and Schwappach, 2018; Inada *et al.*, 2014) to vesicle docking and fusion to the PM (*VfEXO70H7*, *VfSYP43*, and *VfSYP121*; Vukašinovic and Zárský, 2016; Wang *et al.*, 2016; Supplementary Table S7A). At a post-translational level, _apo_H_2_O_2_ could influence vesicle docking through switching on formation of a [Ca^2+^]_cyt_, signal (Zhang *et al.*, 2015*b*) to activate the Ca^2+^-sensitive docking proteins, VfSYT1, VfSYT2, and VfSYT3 (Pérez-Sancho *et al.*, 2016; Supplementary Table S7B). The identified _apo_H_2_O_2_-sensitive docking/fusion proteins are the most probable candidates conferring SED polarity. In the absence of _apo_H_2_O_2_, up-regulated expression of genes encoding endocytotic proteins (*VfHS3P1* and *VfPIP5K2*; Paez Valencia *et al.*, 2016; Supplementary Table S7C) is consistent with _apo_H_2_O_2_ depressing endocytosis.

### The sterol-enriched domain is essential for Ca^2+^ but not _apo_H_2_O_2_ signalling

Lipid-enriched PM domains play key roles in determining the activity and cellular localization of RBOHs across a range of physiological contexts including tip growth of pollen tubes (Liu *et al.*, 2009) and plant–biotroph interactions (Ott, 2017). To this end, we tested whether the _apo_H_2_O_2_ signal of *trans*-differentiating ETCs (Table 1; Fig. 3) was subjected to SED feedback regulation through impacting RBOH activity and/or its intracellular distribution (Andriunas *et al.*, 2012). Pharmacological blockade of SED formation affected neither _apo_H_2_O_2_ biosynthesis nor its intracellular distribution (Table 4; Supplementary Fig. S3).

**Table 4.** Effect of the plasma membrane sterol-enriched domain on _apo_H_2_O_2_ production in *trans*-differentiating epidermal transfer cells of cultured *V. faba* cotyledons

Cotyledon treatment	_apo_H_2_O_2_ flux (pmol mm^−2^ min^−1^)
0 h Control	10.1±0.5
0.5 h Control	33.8±1.3
0.5 h Fenpropimorph (10 µM)	31.6±1.4
0.5 h Myriocin (1 µM)	29.5±1.7

Cotyledons were freshly harvested or cultured on MS medium in the absence or presence of inhibitors of sterol (fenpropimorph) or sphingolipid (myriocin) biosynthesis for 4 h at 4 °C and thereafter for a further 0.5 h at 26 °C to coincide with the peak in _apo_H_2_O_2_ production (Andriunas *et al.*, 2012). Data are means ±SEs from six replicate cotyledons.

The SED could facilitate plumes of elevated [Ca^2+^]_cyt_ being generated in the ETC outer periclinal cytosol by influencing the activity/organization of Ca^2+^-permeable channels and/or Ca^2+^-ATPases in their PM (Zhang *et al.*, 2015*a*). This hypothesis was evaluated by determining whether the magnitude of the [Ca^2+^]_cyt_ signal and/or polarized distribution of DHP-receptor Ca^2+^ channels were sensitive to a pharmacological blockade of SED formation. The blockade was imposed after 9 h of cotyledon culture to ensure that [Ca^2+^]_cyt_ levels had reached a steady state (Table 5; Zhang *et al.*, 2015*b*).

**Table 5.** Effect of the plasma membrane sterol-enriched domain on cytosolic Ca^2+^ concentration ([Ca^2+^]_cyt_) in the outer periclinal region of *trans*-differentiating epidermal transfer cells (ETCs) of cultured *V. faba* cotyledons

Cotyledon treatment	[Ca^2+^]_cyt_ in ETC outer periclinal cytosol (nM)
0 h Control	16±2
9 h Control	652±19
9+6 h Control	630±14
9 h Control+6 h fenpropimorph (10 µM)	17±1
9 h Control+6 h fenpropimorph (10 µM) and Eosin Yellow (500 nM)	16±1
9 h Control+6 h myriocin (1 µM)	17±2
9 h Control+6 h myriocin (1 µM) and Eosin Yellow (500 nM)	19±1

Cotyledons were freshly harvested or cultured on MS medium for 9 h to ensure that [Ca^2+^]_cyt_ had reached a steady state (Zhang *et al.*, 2015*b*), before being transferred to media ±fenpropimorph or ±myriocin overlaid with ±Eosin Yellow, an inhibitor of Ca^2+^-ATPases, for 4 h at 4 °C. Thereafter, cotyledon culture was continued for a further 6 h at 26 °C. [Ca^2+^]_cyt_ levels were then estimated from detected fluorescence levels of OGB-1 loaded into the ETCs. Data are means ±SEs from four replicate cotyledons; 20 cells per cotyledon.

The levels of the polarized [Ca^2+^]_cyt_ signal in ETCs of cotyledons exposed to fenpropimorph or myriocin over the ensuing 6 h were reduced by 37-fold to match those in epidermal cells prior to their entry into the *trans*-differentiation pathway (Table 5; Supplementary Fig. S4). In ETCs, [Ca^2+^]_cyt_ levels are dynamically balanced by Ca^2+^ influx through PM Ca^2+^-permeable channels and efflux through PM Ca^2+^-ATPases (Zhang *et al.*, 2015*a*). In this context, Eosin Yellow inhibition of the Ca^2+^-ATPases (Zhang *et al.*, 2015*a*) did not recover [Ca^2+^]_cyt_ levels in the presence of fenpropimorph or myriocin (Table 5). This finding removes the possibility that an absence of the SED increased Ca^2+^-ATPase activity to account for the reduction in [Ca^2+^]_cyt_ levels. Thus, we concluded that transport activities of PM Ca^2+^-permeable channels were substantially enhanced by being embedded in the SED. This regulatory influence could result from a post-translational activation mechanism and/or regulating their PM residence. The latter possibility was explored as outlined below.

The relative abundance of Ca^2+^-permeable channels was estimated using the fluorescent-tagged nifedipine analogue fl-DHP on the grounds outlined in Zhang *et al.*, (2015*a*). An experimental design identical to that used to obtain estimates of [Ca^2+^]_cyt_ was employed. While total ETC levels of fl-DHP fluorescence remained unchanged, SED deletion between 9 h and 15 h of cotyledon culture led to the intracellular distribution of the nifedipine-sensitive Ca^2+^ channels being depolarized (Table 6; Supplementary Fig. S4). Significantly, channel redistribution was inhibited by BFA, pointing to their endocytosis into the endosome vesicle pathway. Withdrawal of Ca^2+^ channels from the PM would negate any capacity for Ca^2+^ influx into the ETC cytosol from the cell wall compartment to replenish Ca^2+^ pumped in the opposite direction by the Ca^2+^-ATPases and, hence, accounts for the precipitous decline in [Ca^2+^]_cyt_ (Tables 6 and 5, respectively).

**Table 6.** Effect of the plasma membrane (PM) sterol-enriched domain on the distribution of DHP receptor Ca^2+^-permeable channels in *trans*-differentiating epidermal transfer cells (ETCs) of cultured *V. faba* cotyledons

Cotyledon treatment	fl-DHP fluorescence (arbitrary units) in ETC:			
	Outer periclinal PM	Anticlinal PM	Inner periclinal PM	Total
0 h Control	52±4	49±5	51±5	152±8
9 h Control	577±14	52±6	55±6	684±19
9+6 h Control	585±18	54±9	63±6	702±21
9 h Control+6 h fenpropimorph (10 µM)	235±9	226±11	231±12	692±19
9 h Control+6 h myriocin (1 µM)	240±10	227±12	223±12	690±20
9 h Control+6 h fenpropimorph (10 µM) and BFA (357 μM)	569±16	47±5	53±5	669±17
9 h Control+6 h myriocin (1 µM) and BFA (357 μM)	592±17	52±7	56±7	700±20

Cotyledons were cultured on MS medium for 9 h to ensure that the abundance of Ca^2+^-permeable channels had reached a steady state (Zhang *et al.*, 2015*b*), before being transferred to MS medium ±fenpropimorph or ±myriocin with the fenpropimorph and myriocin media containing ±brefeldin (BFA) for 4 h at 4 °C and thereafter cultured for a further 6 h at 26 °C. At the conclusion of culture, the cotyledons were immediately stained with 600 nM DM-BODIPY(–)-dihydropyridine (fl-DHP). Relative abundance of Ca^2+^ channels was estimated from fluorescence levels detected as total pixels in specified regions of the ETC PM. Data at each culture time are means ±SEs from four replicate cotyledons; 20 cells per cotyledon.

### The sterol-enriched domain is essential for wall labyrinth assembly

Comparability of the spatiotemporal enrichment of the SED in the outer periclinal PM region (Figs 1, 2) with that of wall labyrinth assembly in *trans*-differentiating ETCs (Wardini *et al.*, 2007; Xia *et al.*, 2017) suggested that these events may be causally related. This proposition was tested by blocking SED formation on assembly of the UWL and WI papillae.

**Fig. 4.**
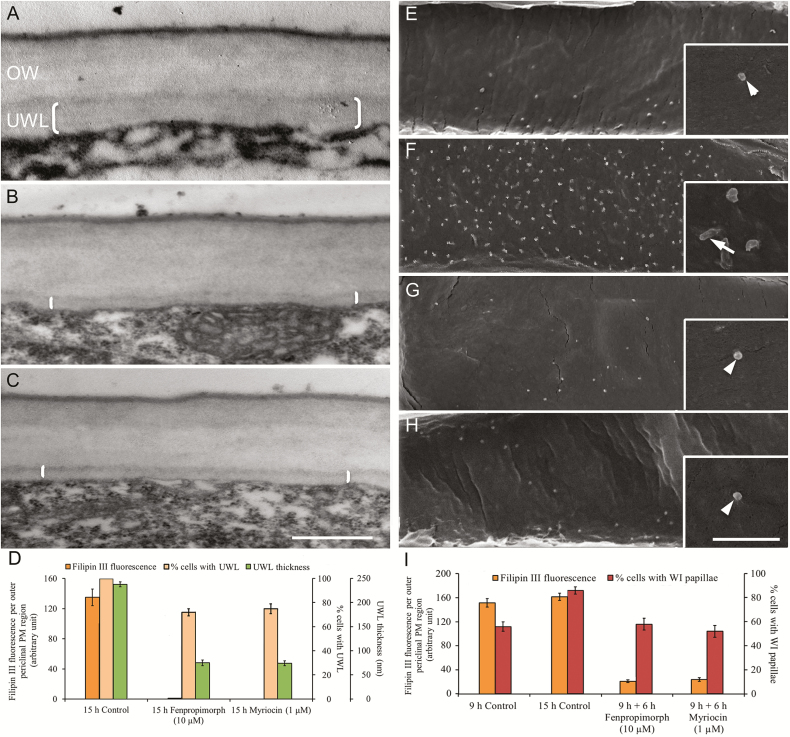
Assembly of (A–D) the uniform wall layer (UWL) and (E–I) wall ingrowth (WI) papillae is dependent on a polarized sterol-enriched domain in the plasma membrane (PM) of *trans*-differentiating epidermal transfer cells (ETCs) of cultured *V. faba* cotyledons. (A–C) TEM images of the outer periclinal wall of ETCs in cotyledons cultured on MS medium in the (A) absence or (B) presence of 10 µM fenpropimorph or (C) 1 µM myriocin at 4 °C for 4 h and thereafter cultured for a further 15 h at 26 °C. The uniform wall layer is indicated by brackets. Scale bar=500 nm. (D) Filipin fluorescence levels detected in outer periclinal PM region of ETCs, percentage ETCs forming a uniform wall layer, and uniform wall layer thickness in cotyledons cultured as described in (A–C). Data are means ±SEs from four replicate cotyledons; 20 cells per cotyledon. (E–H) FESEM images viewed from the cytoplasmic face of the outer periclinal wall of ETCs. Cotyledons were cultured on MS medium for 9 h to ensure deposition of the uniform wall layer (Xia *et al.*, 2017), before (E) being prepared for imaging or (F–H) being transferred to MS in the (F) absence or presence of (G) 10 µM fenpropimorph or (H) 1 µM myriocin at 4 °C for 4 h and thereafter cultured for a further 6 h at 26 °C. Insets show magnified images of WI papillae. Note that further bending and branching of WI papillae (arrow in F) was halted in the presence of the pharmacological inhibitors (arrowheads in G, H). Scale bar=10 µm and 2 µm in insets. (I) Filipin fluorescence levels detected as total pixels in the outer periclinal PM region in, and percentage of, ETCs forming WI papillae in cotyledons cultured as described in (E–H). Data are means ±SEs from four replicate cotyledons, 100 cells (wall ingrowth data) or 20 cells (Filipin data) per cotyledon.

For epidermal cells of cotyledons cultured on fenpropimorph or myriocin, where Filipin fluorescence was reduced 39-fold, ETCs forming a UWL (Fig. 4A–C) decreased from 100% to 75%, while UWL thickness was reduced by 77% (Fig. 4D). To avoid confounding effects of quenching SED formation on UWL assembly (Fig. 4A–D) on the subsequent deposition of WI papillae, exposure of cotyledons to media containing fenpropimorph or myriocin was delayed for the first 9 h of cotyledon culture to ensure that the UWL was fully formed (Xia *et al.*, 2017). Across the intervening 6 h of cotyledon culture on MS medium alone, Filipin fluorescence remained unchanged while percentages of *trans*-differentiating ETCs exhibiting WI papillae increased by 58% (Fig. 4I). In contrast, a Filipin fluorescence decrease by 8- to 9-fold on exposure to fenpropimorph or myriocin was accompanied by cessation of deposition of WI papillae or, for those formed, their bending (Fig. 4F versus Fig. 4G, H versus Fig. 4E) and recruitment of additional epidermal cells to commence assembling WI papillae (Fig. 4I).

Construction of the UWL and WI papillae depends upon polarized vesicle docking to the outer periclinal PM region of *trans*-differentiating ETCs (Dibley *et al.*, 2009; Zhang *et al.*, 2017*a*). In this context, SEDs have been conjectured to facilitate vesicle docking to PMs carrying cargoes of cell wall machinery such as glycosylphosphatidylinositol (GPI)-anchored proteins and enzymes contributing to callose homeostasis in plasmodesmal pores (Iswanto and Kim, 2017). This idea was tested by blocking SED biosynthesis with fenpropimorph or myrocin during construction of the UWL (0–3 h of cotyledon culture; see Xia *et al.*, 2017) or WI papillae (9–12 h of cotyledon culture) alone and monitoring the distribution of vesicles labelled with the membrane fluorescent dye, FM-64FX, at the conclusion of these treatment periods. The findings show that a polarized distribution of FM-64FX fluorescence to the outer periclinal region of the *trans*-differentiating ETCs was dissipated when SED formation was inhibited during formation of the UWL or WI papillae without any impact on total FM-64FX fluorescence per cell (Table 7). The re-distribution of FM-64FX in the absence of the SED can be reversed after removing SED biosynthesis inhibitors. As shown by treating with the endocytosis inhibitor, dynasore, this does not require FM4-64 endocytosed from the PM, consistent with the SED facilitating vesicle docking to the outer periclinal PM domain of the *trans*-differentiating ETCs.

**Table 7.** Effect of the plasma membrane (PM) sterol-enriched domain on vesicle distribution during construction of the (A) uniform wall layer and (B) wall ingrowth papillae in *trans*-differentiating epidermal transfer cells (ETCs) of cultured *V. faba* cotyledons

Cotyledon treatment		FM4-64FX fluorescence (arbitrary units) in ETC:			
		Outer periclinal cytosol	Anticlinal cytosol	Inner periclinal cytosol	Total
(A)	3 h MS	622±16	29±3	32±4	683±17
	3 h Fenpropimorph (10 µM)	231±8	234±6	223±4	688±13
	3 h Fenpropimorph (10 µM)+3 h MS	627±13	31±6	32±4	690±15
	3 h Fenpropimorph (10 µM)+3 h Dynasore (100 µM)	614±12	30±4	33±5	677±15
(B)	12 h MS	414±14	34±6	31±5	479±16
	9 h MS+3 h Fenpropimorph (10 µM)	166±10	159±8	162±8	487±16
	9 h MS+3 h Fenpropimorph (10 µM)+3 h MS	407±14	34±5	35±4	476±16
	9 h MS+3 h Fenpropimorph (10 µM) +3 h Dynasore (100 µM)	411±12	28±4	34±4	473±14

(A) Cotyledons were cultured on MS medium containing the sterol biosynthesis inhibitor, fenpropimorph, for 3 h at 26 °C, then washed 3×5 min in dH_2_O before being transferred to MS medium in the absence/presence of the endocytosis inhibitor, dynasore, for 4 h at 4 °C and subsequently cultured for a further 3 h at 26 °C.

(B) Cotyledons were cultured on MS medium for 9 h to ensure deposition of the uniform wall layer (Xia *et al.*, 2017), before being transferred to MS medium containing fenpropimorph for 3 h at 26 °C, then washed 3×5 min in dH_2_O before being transferred to MS medium in the absence/presence of dynasore for 4 h at 4 °C and subsequently cultured for a further 3 h at 26 °C.

Immediately before and following exposure to dynasore, transverse sections of treated cotyledons were stained with the membrane dye FM4-64FX. Fluorescence was measured as total pixels in specified ETC regions. These values were adjusted for fluorescence from FM4-64FX located in the PM using the BFA values (Zhang *et al.*, 2017*a*) to provide estimates of cytoplasmic fluorescence. Data are the mean differences ±SE between mean pixel levels of four replicate cotyledons; 20 cells per cotyledon.

## Discussion

Upon transfer of *V. faba* cotyledons to culture, the PM in the outer periclinal portion of their adaxial *trans*-differentiating ETCs progressively became enriched in sterols that asymptote to reach an ongoing steady-state level within 9 h (Fig. 2; Supplementary Fig. S2). The polarized ETC SEDs corresponded to those formed in the outer planar domain of the PM of root epidermal cells preceding root hair initiation (e.g. Stanislas *et al.*, 2015). While localized SEDs have been observed in a number of physiological contexts including tips of elongating root hairs and pollen tubes (Ovecka *et al.*, 2010; Liu *et al.*, 2009), sites of defence papillae (Faulkner, 2015), and plasmodesmal construction (Iswanto and Kim 2017), little is known about the upstream mechanisms regulating their formation and maintenance (Han *et al.*, 2018). Our ETC cotyledon induction system presented the opportunity of exploring this question as well as unravelling how SEDs may function as a PM-located platform to organize signals regulating machinery constructing the ETC wall labyrinth.

### Ethylene and hydrogen peroxide signals co-regulate assembly of a polarized sterol-enriched domain

**Fig. 5.**
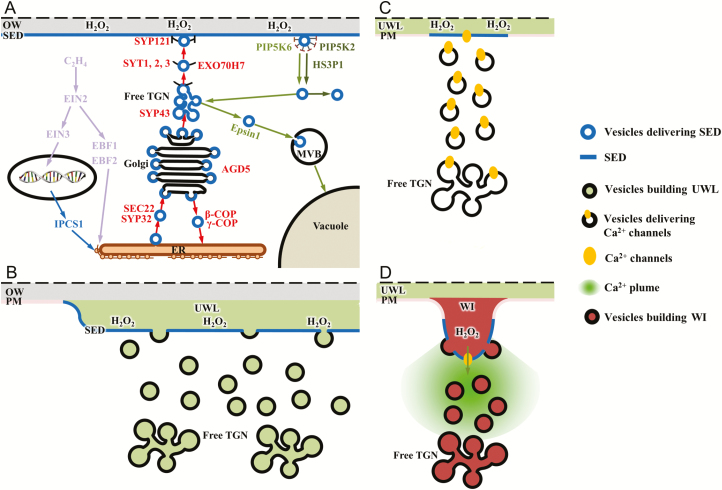
Schematic model illustrating the (A) formation and (B–D) role of the sterol-enriched domain (SED) in the outer periclinal region of the plasma membrane (PM) of *trans*-differentiating epidermal transfer cells (ETCs) of *V. faba* cotyledons. (A) Impact of ethylene and _apo_H_2_O_2_ on biosynthesis and intracellular localization of the SED. Key genes encoding proteins involved in SED biosynthesis and vesicle trafficking (exo/endocytosis) sensitive to ethylene and _apo_H_2_O_2_ are listed (for more details, see Supplementary Tables S5 and S7). Proteins stimulated and blocked by ethylene are highlighted in blue and purple, respectively. Proteins stimulated and blocked by _apo_H_2_O_2_ are highlighted in red and green, respectively. Endoplasmic reticulum (ER); multivesicular body/late endosome (MVB); original wall (OW); *trans*-Golgi network (TGN); the dashed line, indicates incomplete outer periclinal wall width. (B) Regulated by _apo_H_2_O_2_, the SED provides an as yet to be defined microenvironment in the PM for selective docking and exocytosis of vesicles carrying cargoes to construct the uniform wall layer (UWL). (C) On completion of UWL formation, and under _apo_H_2_O_2_ regulation, the SED is envisaged to re-aggregate into clusters throughout the PM that define loci for the docking and fusion of vesicles carrying Ca^2+^-permeable channels. (D) The Ca^2+^ channels generate plumes of elevated [Ca^2+^]_cyt_ that remodel the actin network to direct flow of vesicles carrying cargoes to construct WI papillae at the [Ca^2+^]_cyt_ plume-defined loci.

The spatial and temporal formation of the SED in the *trans*-differentiating ETCs coincided with construction of their wall labyrinth (Wardini *et al.*, 2007; Xia *et al.*, 2017). Therefore, it was not surprising to discover that these two developmental events were orchestrated by some of the same regulatory signals, specifically ethylene and _apo_H_2_O_2_ (Table 1; Andriunas *et al.*, 2013). In contrast to inhibiting transcription of genes encoding enzymes in the sphingolipid biosynthesis pathway of Arabidopsis seedlings (Wu *et al.*, 2015), ethylene activated phytosterol biosynthesis in the ETCs (Table 1). This was mediated by ethylene acting at the transcriptional (16%) and translational (77%) levels (Table 2). Translational control by ethylene (Fig. 5A) could occur through the recently described cytosolic action of EIN2 via EBF1 and EBF2 (Salehin and Estelle, 2015; Zheng and Zhu, 2016) as indicated by their up-regulated expression in the absence of ethylene. A downstream target for cytosolic EIN2 action could be the translation of *VfHMG-CoA synthase1* (Supplementary Table S5A), the second enzyme in the mevalonate pathway, known to be regulated at the transcriptional, translational, and post-translational levels (Liao *et al.*, 2018). Transcriptional regulation by ethylene of wall labyrinth assembly is considered to occur through an ETC-specific ethylene signalling pathway under control of the nuclear-located transcription factors EIN3 and its homologue EIN3-like (Zhou *et al.*, 2010). Surprisingly, ethylene-regulated expression of sterol biosynthetic genes was not detected. Rather, ethylene up-regulated expression of *VfIPCS1* (Fig. 5A), encoding a key enzyme in the biosynthetic pathway generating the largest group of plant sphingolipids, glycosyl inositol phosphoceramides (Michaelson *et al.*, 2016). A tight relationship existed between phytosterol and sphingolipid biosynthesis, as illustrated by the pharmacological blockade of sphingolipid biosynthesis resulting in lowered ETC levels of phytosterols (Fig. 2). This linkage could be mediated through HMG-CoA reductase that is known to be a site for co-ordinated post-translational regulation between sterol and sphingolipid biosynthesis (Nieto *et al.*, 2009).

The polarized flow of phytosterols from their sites of synthesis in the endoplasmic reticulum (ER)/Golgi to the outer periclinal portion of the ETC PM was directed by an _apo_H_2_O_2_ signal (Table 1; Fig. 3) localized to the outer periclinal wall of ETCs (Fig. 5A; Andriunas *et al.*, 2012). The SED polarization was independent of the microtubule and actin cytoskeletons (Table 3), a feature that departs from the cytoskeleton-dependent polarity of sterol-enriched membrane domains in animal cells (Byrum and Rodgers, 2015). The cytoskeletal independence points to _apo_H_2_O_2_ activating a sterol-specific docking/fusion mechanism in the ETC PM. Possibilities include docking/fusion of sterol-enriched secretory vesicles or sterol-specific lipid transfer proteins that ferry sterols across membrane contact sites between the ER and PM (Pérez-Sancho *et al.*, 2016; Wang *et al.*, 2017). Some hints as to the mechanism were deduced from profiling the ETC-specific transcriptome for _apo_H_2_O_2_-dependent expression of DEGs encoding candidate proteins.

In support of secretory vesicle docking/fusion, expression of genes encoding the PM-localized Qa-SNARE homologues, VfSYP43 and VfSYP121, and an exocyst subunit, VfEXO70H7, was found to be _apo_H_2_O_2_ dependent (Fig. 5A). SYP43 plays a number of roles at the *trans-*Golgi network that include directing vesicles decorated with the R-SNARE, VAMP721/722, to the PM for docking/fusion mediated by PM-localized SYP121 (Wang *et al.*, 2016). Significantly, SYP121 interactions direct tip growth of root hairs (Ichikawa *et al.*, 2014) and localized formation of defence papillae at contact sites with biotrophic pathogens (Faulkner, 2015). Subunits of EXO70 play similar roles in polarized growth systems (Vukašinovic and Zárský, 2016). At a post-translational level, _apo_H_2_O_2_ could contribute to polarized vesicle docking through switching on a polarized [Ca^2+^]_cyt_ signal (Zhang *et al.*, 2015*b*) that in turn activated Ca^2+^-sensitive tethering synaptotagmin (SYT) proteins, VfSYT1, VfSYT2, and VfSYT3 (Pérez-Sancho *et al.*, 2016; see Fig. 5A), a function linked with tip growth of pollen tubes (Wang *et al.*, 2015). Expression of DEGs encoding sterol-specific lipid transfer proteins was found to be insensitive to _apo_H_2_O_2_, leaving open whether the ER to PM pathway is operative in *trans*-differentiating ETCs.

The rapid _apo_H_2_O_2_-dependent assembly and disassembly of SEDs in the ETC PM (Fig. 3) is suggestive of endocytosis and vesicle recycling through the early endosome pathway, a role proposed in establishing pollen tube polarity (Guo *et al.*, 2012). Consistent with this suggestion, absence of _apo_H_2_O_2_ caused up-regulated expression of genes encoding homologues of the endocytotic proteins, VfPIP5K2 and VfSH3P1 (Fig. 5A). Recruitment of factors to the PM driving clathrin-mediated endocytosis depends upon the synthesis of phosphatidylinositol-4,5-bisphosphate catalysed by PIP5K2, while HS3P1 facilitates disassembly of the clathrin coat as a prelude to endocytosed vesicle fusion with endosomes (Paez Valencia *et al.*, 2016). Thus, similar to auxin regulation of PIN proteins at the PM (Paez Valencia *et al.*, 2016), _apo_H_2_O_2_ exerted a positive effect on SED vesicle docking/fusion with the ETC PM and acted negatively to prevent SED endocytosis to stabilize the SED at the PM.

In contrast to the AA-induced endocytotic withdrawal of SEDs from the PM into early endosomal vesicles that are re-distributed equally around the ETC cytosol, the SEDs were completely dismantled in the absence of ethylene (Fig. 5A; Supplementary Table S4). The response to an absence of ethylene has two components. First, AVG is known to inhibit _apo_H_2_O_2_ production by ~40% (Andriunas *et al.*, 2012) with a consequent enhanced endocytosis of SED-containing vesicles from the PM (see above). This effect would be further amplified by the AVG-induced de-repression of *VfPIP5K6* expression that functions in clathrin-mediated endocytosis (Zhao *et al.*, 2010). The enhanced endocytosis in the absence of ethylene was coupled with a strong up-regulated expression of *VfEpsin1* known to direct vesicle trafficking to vacuolar lysis (Fig. 5A; Song *et al.*, 2006).

### Generation of calcium, but not hydrogen peroxide, signals is dependent on the sterol-enriched domain

In contrast to other plant cell systems (e.g. Liu *et al.*, 2009; Hao *et al.*, 2014), the presence of SEDs in the ETC PM did not influence the formation, or intracellular distribution, of their polarized _apo_H_2_O_2_ signal (Table 4; Supplementary Fig. S3), putatively generated by PM-localized RBOHs (Andriunas *et al.*, 2012). An explanation for _apo_H_2_O_2_ signal formation being independent of the PM SED could be that the signal is generated by diphenyleneiodonium chloride-sensitive flavin-containing enzymes located in the cell wall, such as class III peroxidases found in elongating root hairs (Mangano *et al.*, 2017).

Using responses of the polarized plumes of elevated [Ca^2+^]_cyt_ formed in the *trans*-differentiating ETCs as a proxy to interpret SED regulation of the PM-located Ca^2+^-permeable channels and Ca^2+^-ATPase activities (Zhang *et al.*, 2015*a*), we concluded that the SEDs selectively regulated the activity of the co-localized Ca^2+^-permeable channels (Tables 5, 6; Fig. 5C). To our knowledge, this is the first reported presence of Ca^2+^ channels in a PM SED of a plant cell. However, such associations are well established for animal cells (Pani and Singh, 2009). Nevertheless, proteomic analyses of sterol-dependent proteins in detergent-resistant membranes of mature pollen grains detected a suite of Ca^2+^ signalling proteins including Ca^2+^-ATPases (Han *et al.*, 2018).

The sterol-dependent activity of the ETC Ca^2+^-permeable channels appeared to be an all or none response achieved by their endocytosis into the endosome vesicle pathway in the absence of the SED in the ETC PM (Table 6). The sterol independence of Ca^2+^-ATPase activity, that also occupies the outer periclinal region of the ETC PM, could be explained by their spatial inter-relationship with the Ca^2+^-permeable channels. Within this ETC region, the Ca^2+^-permeable channels are envisaged to be organized in clusters at the tips of developing WI papillae surrounded by a matrix of Ca^2+^-ATPases located between the WI papillae (Zhang *et al.*, 2015*a*). This predicts that the SEDs are localized to WI papillae tips (Fig. 5D) where exo- and endocytosis are considered to be most active (Zhang *et al.*, 2017*a*). Thus, blockading SED formation, by inhibiting sterol/sphingolipid biosynthesis, prevents vesicles carrying the Ca^2+^-permeable channels docking with the tips of developing WI papillae (Fig. 5C). As a consequence, endocytosis predominates, with endocytosed vesicles carrying the Ca^2+^-permeable channels being re-distributed equally around the ETC cytosol (Table 6).

### The sterol-enriched domain is essential for wall labyrinth formation

The SED-dependent assembly of the polarized ETC wall labyrinth (Fig. 5B, D) joins that found for maintenance of polarized tip growth of root hairs and pollen tubes (Liu *et al.*, 2009; Ovecka *et al.*, 2010) and assembly of defence papillae (Faulkner, 2015). However, in the case of ETC wall labyrinth formation, the very disparate assembly patterns of the UWL and WI papillae (Xia *et al.*, 2017) suggest that SED composition/spatial organization must undergo substantive shifts as wall labyrinth formation progresses from UWL to WI papillae construction. In the case of SED composition, there is a shift in the expression of genes encoding sterol biosynthesis between the phases of wall labyrinth formation (see Supplementary Table S5B) that could impact vesicle trafficking and PM properties (Pook *et al.*, 2017; Sena *et al.*, 2017). Moreover, UWL formation is characterized by an even deposition of matrix polysaccharides across the outer periclinal original wall and is independent of cellulose biosynthesis. The reverse applies to the cellulose-dominated assembly of WI papillae arising from loci on the cytoplasmic face of the UWL (Xia *et al.*, 2017). Thus, the cytoskeleton-independent assembly of the UWL (Xia *et al.*, 2017; Zhang *et al.*, 2017*a*) must rely on ethylene/_apo_H_2_O_2_-regulated formation of the SED (as described earlier) to provide a platform for docking and exocytosis of vesicles carrying cargoes of matrix polysaccharides (Fig. 5B). In contrast, the SED responsible for WI papillae formation must be localized in clusters, ~500 nm in diameter (Zhang *et al.*, 2017*a*), in the ETC PM (Fig. 5D). While the _apo_H_2_O_2_ signal continues to define the outer periclinal domain for SED incorporation during WI papillae assembly (Fig. 4), whether the SED micro pattern in the outer periclinal region is solely governed by _apo_H_2_O_2_ remains to be determined. The SED clusters define sites for insertion of the Ca^2+^-permeable channels (Fig. 5C). These channels create cytosolic plumes of elevated [Ca^2+^]_cyt_ that remodel the actin network to deliver vesicles, carrying cargoes of cell wall materials, to the Ca^2+^-defined loci for WI papillae assembly (Fig. 5D; Zhang *et al.*, 2017*a*). In addition, the predicted differing sterol composition of the SED between the UWL and WI papillae phases of wall labyrinth formation (Supplementary Table S5B) could impact cellulose biosynthesis activities (Schrick *et al.*, 2012). The proposed sterol regulation might contribute to the lowered cellulose content of the UWL (Xia *et al.*, 2017) and to the dense whorls of cellulose microfibrils essential for WI papillae formation (Talbot *et al.*, 2007).

## Supplementary data

Supplementary data are available at *JXB* online.

Fig. S1. Filipin staining co-localizes with the PM of ETCs.

Fig. S2. Temporal pattern of SED formation.

Fig. S3. Effect of the SED on _apo_H_2_O_2_ distribution.

Fig. S4. Effect of the SED on Ca^2+^ signalling and Ca^2+^ channel distribution

Table S1. Lengths of specified PM domains in ETCs and SPCs.

Table S2. Effect of blocking vesicle trafficking on SED distribution.

Table S3. Effect of sphingolipid, sterol, and brassinosteroid inhibitors on SED formation.

Table S4. Levels of biological control exercised by ethylene on SED formation.

Table S5. Impact of AVG on transcript abundance of sphingolipid- and phytosterol-related genes.

Table S6. Effect of vesicle trafficking on the regulation of sterol distribution by _apo_H_2_O_2_ signalling.

Table S7. Impact of ascorbic ascid on transcript abundance of vesicle trafficking genes.

Supplementary Data

## Abbreviations

AA: ascorbic acid
ACC: 1-aminocyclopropane-1-carboxylic acid
_apo_H_2_O_2_: extracellular hydrogen peroxide
AVG: aminoethoxyvinylglycine
DAB: 3',3'-diaminobenzidine
BFA: brefeldin A
[Ca^2+^]_cyt_: cytosolic calcium
fl-DHP: DM-BODIPY(–)-dihydropyridine
EBF: ethylene-binding F-box protein
ETC: epidermal transfer cell
LatB: latrunculin B
MS: Murashige and Skoog
OGB-1: Oregon Green BAPTA-1 acetoxymethyl ester
PCIB: *p*-chlorophenoxyisobutyric acid
PM: plasma membrane
SED: sterol-enriched domain
SPC: storage parenchyma cell
TC: transfer cell
UWL: uniform wall layer
WI: wall ingrowth.
