# Supplementary material for: Ethylene and hydrogen peroxide regulate formation of a sterol-enriched domain essential for wall labyrinth assembly in transfer cells
Source: J Exp Bot. 2019 Jan 14;70(5):1469–82. doi: 10.1093/jxb/erz003 (PMC6411373; doi:10.1093/jxb/erz003)
Supplement: Supplementary Data [file erz003_suppl_supplementary_data.pdf]

Supplementary Data

Ethylene and Hydrogen Peroxide Regulate Formation of a Sterol-Enriched Domain Essential for Wall Labyrinth Assembly in Transfer Cells

Hui-Ming Zhang, Luke B. Devine, Xue Xia, Christina E. Offler**and John W. Patrick **

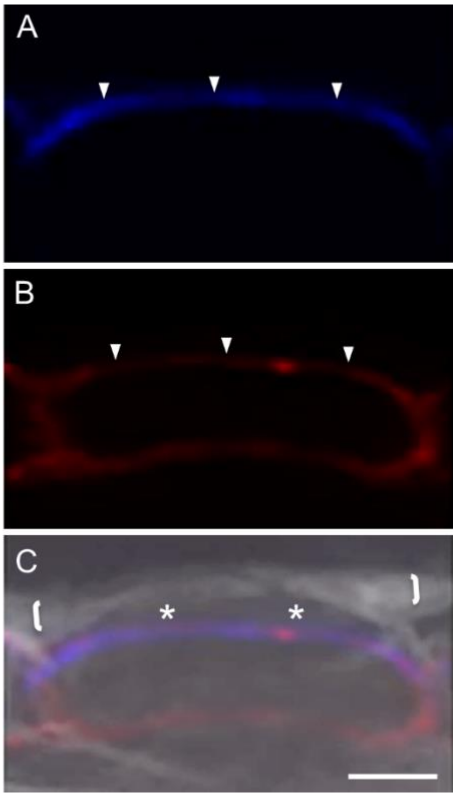

Supplementary Figure S1. Filipin staining co-localized with the plasma membrane (PM) of plasmolyzed *trans*-differentiating epidermal transfer cells (ETCs) of cultured *V. faba* cotyledons.

CLSM images of transverse sections of plasmolyzed ETCs stained with Filipin to detect sterol-enriched domains (fluorescence indicated by darts in A) and the PM tracker RH-414 (fluorescence indicated by darts in B) and. Image overlay of the RH-414 and Filipin co-stained ETC (C) with its outer periclinal wall delimited by brackets and the plasmolytic space between the cell wall and plasma membrane indicated by asterisks. Scale bar = 5 µm.

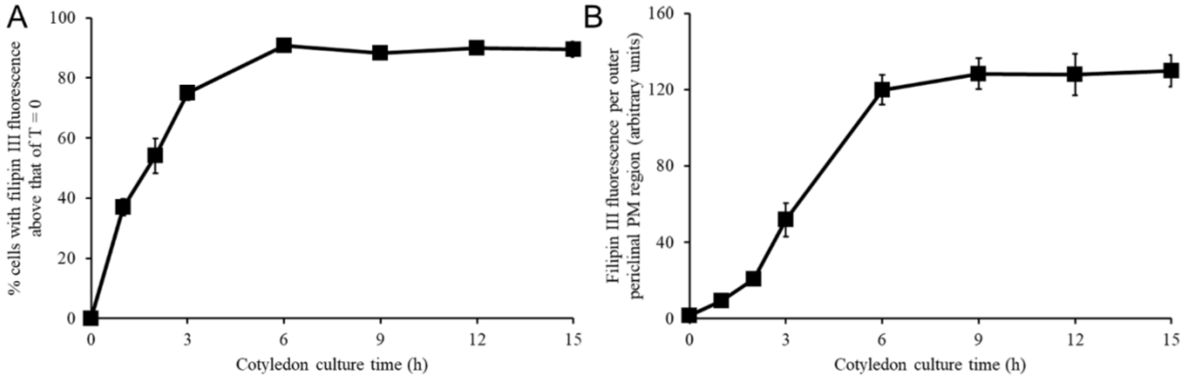

Supplementary Figure S2. Temporal pattern of sterol-enriched domain (SED) formation in the outer periclinal plasma membrane (PM) region of *trans*-differentiating epidermal transfer cells (ETCs) of cultured *V. faba* cotyledons.

Cotyledons were cultured on MS medium for specified times at 26°C. Thereafter, transverse sections of treated cotyledons were stained with Filipin. Fluorescence was measured as total pixels in the outer periclinal PM region of the ETCs. (A) Percentages of ETCs with detectable levels of Filipin fluorescence above that detected at T = 0 h. (B) Relative abundance of SEDs deduced from Filipin fluorescence levels detected as total pixels in the outer periclinal PM region. Data are Means ± SEs from four replicate cotyledons; 50 cells (A) or 20 cells (B) per cotyledon.

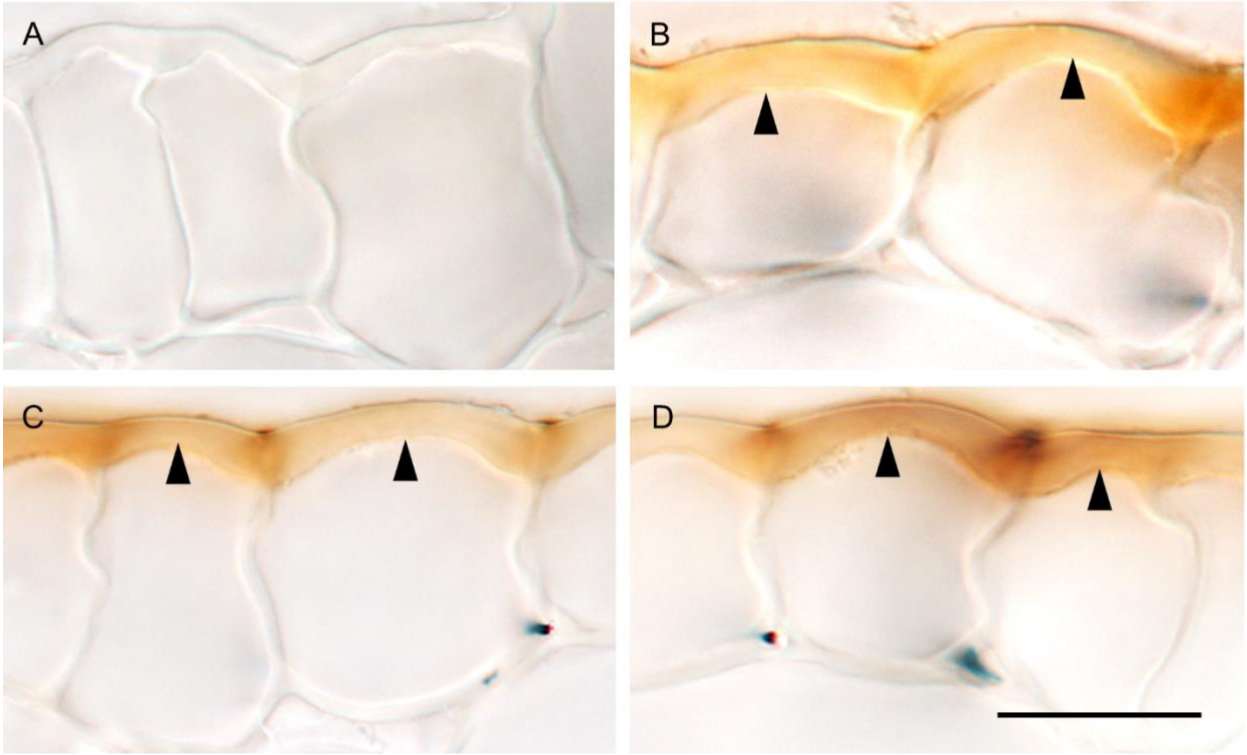

Supplementary Fig. S3. Effect of the sterol-enriched domain (SED) on intracellular $_{apo}H_2O_2$ distribution in *trans*-differentiating epidermal transfer cells (ETCs) of cultured *V. faba* cotyledons.

Micrographs of transverse sections of ETCs showing histochemical detection by DAB of the cellular distribution $_{apo}H_2O_2$. Excised cotyledons were (A) freshly harvested or placed on MS medium in the (B) absence or (C) presence of 10 µM fenpropimorp or (D)1 µM myriocin for 4 h at 4 °C. Thereafter, cotyledons were cultured on MS medium ± 10 µM fenpropimorph or myriocin at 26 °C for another 2 h at 26 °C in freshly prepared MS medium containing 5 mg/mL DAB at pH 5.8. Cotyledons were then sectioned for imaging. DAB-labelled $_{apo}H_2O_2$ signals are marked by darts. Scale bar =10 µm.

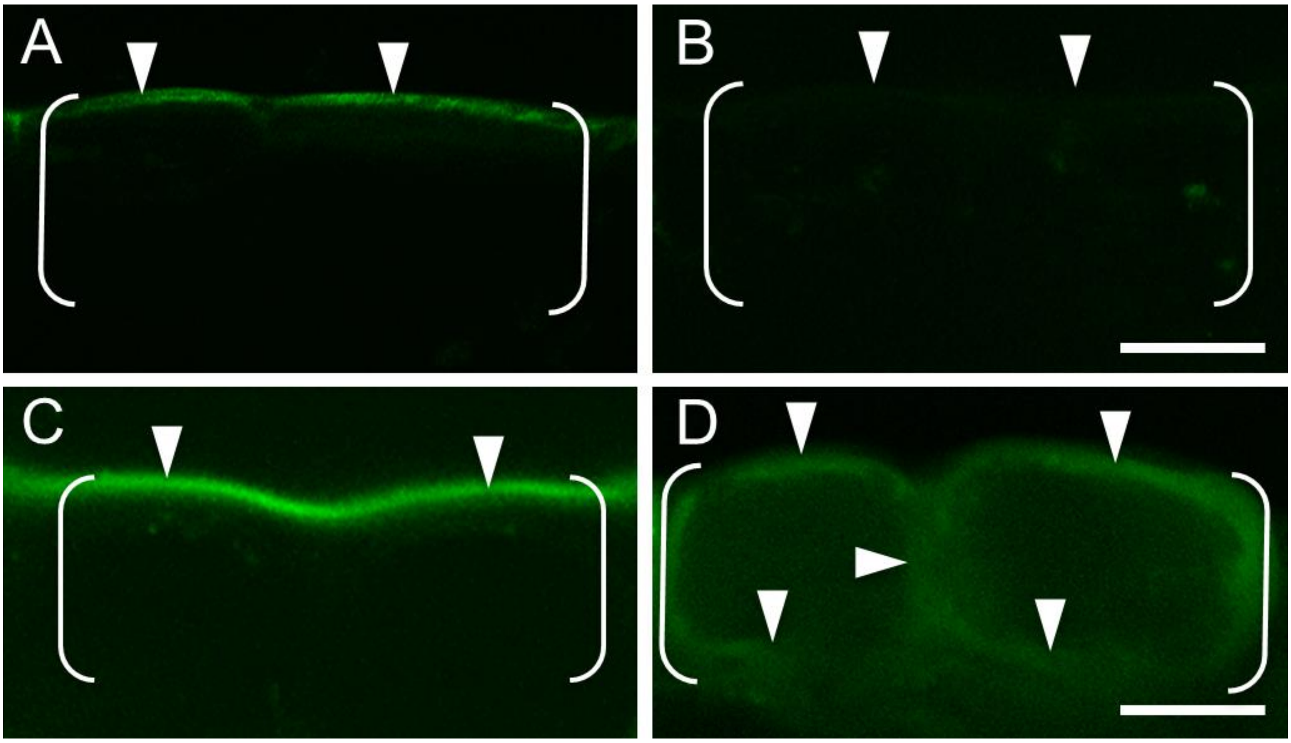

Supplementary Figure S4. Effect of the plasma membrane sterol-enriched domain on cytosolic Ca^{2+} signalling and the distribution of DHP-receptor Ca^{2+}-permeable channels in *trans*-differentiating adaxial epidermal transfer cells (ETCs, bracketed) of cultured *V. faba* cotyledons.

Cotyledons were cultured on MS medium for 9 h to ensure that $[Ca^{2+}]_{cyt}$ and the abundance of Ca^{2+}-permeable channels had reached a steady state (Zhang *et al.*, 2015b), before being transferred to media in the (A, C) absence or (B, D) presence of 10 µM fenpropimorph for 4 h at 4 ^{o}C. Thereafter, cotyledon culture was continued for a further 6 h at 26 ^{o}C. The cultured cotyledons were then either (A, B) loaded with 20 µM Ca^{2+}-sensitive dye, Oregon Green BAPTA-1 acetoxymethyl (AM) ester (OGB-1) to detect $[Ca^{2+}]_{cyt}$ in the ETCs or (C, D) immediately stained with 600 nM DM-BODIPY(–)-dihydropyridine (fl-DHP) to label the DHP-receptor Ca^{2+}-permeable channels in the ETCs. Note that an elevated $[Ca^{2+}]_{cyt}$ was localized to the outer periclinal cytosol (darts in A) of the ETCs and was absent in the presence of fenpropimorph (B) while the intracellular distribution of fl-DHP fluorescence was depolarized in the presence of fenpropimorph (darts in D cf C). Bar = 10 µm.

Supplementary Table S1. Effect of blocking vesicle trafficking on the sterol-enriched domain (SED) distribution in the plasma membrane (PM) of *trans*-differentiating epidermal transfer cells of cultured *V. faba* cotyledons.

Cotyledons were cultured on MS medium for 9 h, before being imaged or transferred to media with or without the vesicle trafficking inhibitor, brefeldin A (BFA) and held at 4 ^{o}C for 4 h. Thereafter the cotyledons were cultured for a further 6 h at 26°C. Following culture, transverse sections of treated cotyledons were stained with Filipin. Fluorescence was measured as total pixels in specified plasma membrane regions of the ETCs. Data are Means ± SEs from four replicate cotyledons; 20 cells per cotyledon.

Cotyledon treatment	Filipin fluorescence (arbitrary units) in the:			
	Outer periclinal PM	Anticlinal PM	Inner periclinal PM	Total
9 h Control	137 ± 10	8 ± 2	1 ± 0	146 ± 11
15 h Control	128 ± 8	3 ± 1	2 ± 1	133 ± 9
9 h Control + 6 h BFA (357 µM)	123 ± 5	5 ± 2	0 ± 0	128 ± 6

Supplementary Table S2. Lengths of specified plasma membrane (PM) regions in *trans*-differentiating epidermal transfer cells (ETCs) and storage parenchyma cells (SPCs) of cultured *V. faba* cotyledons.

Cotyledons were freshly harvested or cultured on MS medium containing ± fenpropimorph or ± myriocin for 15 h. Data are Means ± SEs from four replicate cotyledons; 20 cells per cotyledon for ETCs and 10 cells for SPCs.

Cotyledon treatment	PM lengths (µm) in specified regions of transverse sections of ETCs and SPCs:					
	Outer periclinal		Anticlinal		Inner periclinal	
	ETC	SPC	ETC	SPC	ETC	SPC
0 h Control	19.1 ± 0.4	59.2 ± 3.7	18.4 ± 0.7	56.6 ± 1.5	19.8 ± 0.7	57.7 ± 2.9
15 h Control	19.4 ± 0.7	61.8 ± 3.5	18.4 ± 0.5	58.7 ± 2.2	20.1 ± 0.7	60.1 ±3.8
15 h Fenpropimorph (10 µM)	20.2 ± 0.7	62.4 ± 2.6	19.1 ± 0.6	57.9 ± 2.3	19.5 ± 0.3	59.7 ± 3.1
15 h Myriocin (1 µM)	19.3 ± 0.6	60.8 ± 3.3	18.2 ± 0.5	58.8 ± 1.8	19.2 ± 0.6	58.6 ± 2.2

Supplementary Table S3. Effect of sphingolipid, sterol and brassinosteroid inhibitors on sterol-enriched domain (SED) formation in the outer periclinal region of the plasma membrane (PM) of *trans*-differentiating epidermal transfer cells (ETCs) of cultured *V. faba* cotyledons.

Cotyledons were freshly harvested or cultured on MS medium in the absence or presence of inhibitors of sphingolipid (fumonsin B1) or brassinosteroid (triadimefon and propiconazole) biosynthesis for 4 h at 4°C and thereafter for a further 15 h at 26°C. In a separate experiment, cotyledons were cultured in MS medium for 14 h, then transferred to MS medium containing sterol stripping reagent, methyl-ß -cyclodextrin and cultured for a further 60 min. Thereafter, transverse sections of treated cotyledons were stained with Filipin. Fluorescence was measured as total pixels in the outer periclinal PM region of ETCs. Data are Means ± SEs from four replicate cotyledons; 20 cells per cotyledon.

Cotyledon treatment	Filipin fluorescence (arbitrary units/outer periclinal PM region)
Control	143 ± 11
Fumonsin B1 (1 µM)	8 ± 7
Triadimefon (10 µM)	134 ± 10
Propiconazole (10 µM)	138 ± 13
Methyl-ß -cyclodextrin (10.3 mM)	17 ± 10

Supplementary Table S4. Effect of ethylene on sterol formation in *trans*-differentiating epidermal transfer cells (ETCs) of cultured *V. faba* cotyledons when transcriptional or translational activity was blocked.

Cotyledons were cultured on MS medium alone for 9 h by which time Filipin fluorescence levels had reached a steady-state (Supplementary Fig. S2B). Thereafter cotyledons were transferred to MS medium containing aminoethoxyvinylglycine (AVG – ethylene absent) or AVG plus the ethylene precursor, 1-aminocyclopropane-1-carboxylic acid (ACC - ethylene restored). These treatments were overlaid with exposure of cotyledons to (1) MS media alone or (2) MS medium containing the RNA biosynthesis inhibitor, 6-methylpurine, that restricted ethylene action to the translational plus post-translational levels or (3) a protein biosynthesis inhibitor, cycloheximide, which isolated any regulation by ethylene to the post-translational level. Following 4 h at 4°C, cotyledon culture was continued for a further 6 h at 26°C. In each of these treatments, the role of ethylene on sterol biosynthesis was evaluated by measuring total Filipin fluorescence as total pixels/ETC. Data are Means ± SEs from four replicate cotyledons; 20 cells per cotyledon.

Transcription/ translation inhibitor	Total Filipin fluorescence/ETC (arbitrary units) under conditions of:	
	Ethylene absence (100 µM AVG)	Ethylene presence (100 µM AVG + 100 µM ACC)
(1) Control	5 ± 1	211 ± 10
(2) 6-methyl purine (100 µM)	9 ± 2	182 ± 8
(3) Cyclohexamide (100 µM)	4 ± 1	18 ± 3

Supplementary Table S5. Impact of the ethylene biosynthesis inhibitor, AVG, on transcript abundance of (A) up-regulated epidermal transfer cell (ETC) specific differentially expressed genes (DEGs) encoding sphingolipid (green text)/phytosterol (blue text) biosynthetic enzymes, (B) expressed genes (RPKM>1) encoding proteins sensitive to cytosolic EIN-2, and (C) down-regulated ETC specific DEGs encoding proteins involved in endocytosis/late endosomal lysis pathway promoted by AVG. ETC-specific DEGs met the following criteria: Number of uniquely mapped reads per kilo base per million reads (RPKM) of transcript in ETCs at 3 h or 12 h > 1; 2-fold change (Log2 FC) of > 1 with a false discovery rate (FDR) corrected P value < 0.05 calculated using LimmaR (Ritchie ME, Phipson B, Wu D, Hu Y, Law CW, Shi W, Smyth GK.2015. limma powers differential expression analyses for RNA-sequencing and microarray studies. Nucleic Acids Research 43, e47.) between either 0 to 3 h (uniform wall layer specific DEGs), 3 to 12 h (wall ingrowth papillae specific DEGs) or sustained up-regulation from 0 to 3 h to 12 h of cotyledon culture (uniform wall layer and wall ingrowth papillae shared DEGs); DEGs detected in the ETCs were absent from the storage parenchyma cells (SPCs). In specified cases, where transcript abundance of genes was significantly impacted by AVG (P<0.05), these responses are presented as percentile changes compared to the control. Function of the encoded protein was inferred by the best-fit percentage amino acid alignment with the closest Arabidopsis homolog using TAIR10 and Araport 11 databases. RPKM values are Means ± SE of 6 replicate batches of cotyledons in ETCs and 3 replicates in SPCs. DEGs indicated by asterisks.

Sequence ID	Name	Relative transcript level (RPKM) in:						Log$_2$ fold change in:				Sensitivity to AVG at:	
		ETCs at:			SPCs at:			ETCs at:		SPCs at:			
		0 h	3 h	12 h	0 h	3 h	12 h	0 to 3 h	3 to 12 h	0 to 3 h	3 to 12 h	3 h	12 h
**(A) Up-regulated ETC-specific DEGs**													
**Uniform wall layer specific**													
CL7393.C2	*VfLCB2 serine palmitoyltransferase 1 (CB2SPT1)*	2.2 ± 0.2	7.3 ± 0.7	3.4 ± 0.3	1.6 ± 0.0	4.1 ± 1.1	3.6 ± 0.5	1.7*	-1.1*	1.4	-0.2	No impact	No impact
CL9280.C2	*Vf Serine Palmitoyltransferase 2 (SPT2)*	19.4 ± 0.4	50.3 ± 1.9	29.1 ± 0.7	9.9 ± 0.6	14.7 ± 0.7	11.4 ± 1.0	1.4*	-0.8	0.6	-0.4	No impact	No impact
U19699	*VfSphingoid Long-chain base hydroxylase (SBH)*	9.7 ± 0.5	100.5 ± 11.3	13.2 ± 0.5	5.6 ± 0.3	8.8 ± 0.6	8.9 ± 0.8	3.4*	-2.9*	0.7	0.0	No impact	No impact
CL5664.C1	*Vfinositol phospoceramide synthase1(IPCS1)*	5.8 ± 0.5	29.3 ± 2.5	7.2 ± 0.5	2.3 ±0.0	8.3 ± 0.5	10.7 ± 2.7	2.3*	-2*	1.9	0.4	-23%	No impact
U21000	*VfΔ8-sphingolipid desaturase (Δ^8-SD)*	66.0 ± 1.4	231.1 ± 10.5	93.9 ± 5.4	61.2 ± 5.0	94.2 ± 2.6	149.7 ± 9.6	1.8*	-1.3*	0.6	0.7	No impact	No impact
**Wall ingrowth papillae specific**													
U26936	*Vf3-ß hydroxysteroid dehydrogenase/isomerase (3ßHSD)*	35.1 ± 2.0	13.8 ± 0.5	28.0 ± 2.6	6.8 ± 1.6	11.4 ± 2.7	9.3 ± 1.5	-1.3*	1.0*	0.8	-0.3	20%	No impact
U20624	*VfNAD(P)-binding Rossmann-fold superfamily protein (TSC10B)*	25.0 ± 0.5	11.9 ± 0.9	28.0 ± 1.0	23.8 ± 0.8	28.7 ± 3.8	21.8 ± 1.8	-1.1*	1.2*	0.3	-0.4	No impact	No impact
**Shared uniform wall layer and wall ingrowth papillae**													
U9772	*Vf3-HYDROXY-3-METHYLGLUTARYL-COA SYNTHASE1(HMGS1)*	34.5 ± 1.4	100.8 ± 10.1	93.2 ± 6.7	26.3 ± 2.4	40.0 ± 1.9	46.1 ± 3.7	1.5*	-0.1	0.6	0.2	No impact	No impact
CL4389.C3	*VfNeutral ceramidase (NCER)*	33.3 ± 1.6	176.0 ± 17.2	215.7 ± 28.9	8.2 ± 0.0	14.2 ± 0.5	14.9 ± 0.4	2.4*	0.3	0.8	0.1	No impact	No impact
**(B) Expressed genes sensitive to cytosolic EIN-2**													
U25685	*Vfethylene binding factor1 (EBF1)*	14.2 ± 0.9	17.9 ± 1.1	20.3 ± 0.9	3.3 ± 0.3	10.2 ± 0.5	7.8 ± 0.8	0.3	0.2	1.6*	-0.4	No impact	18%
U23934	*Vfethylene binding factor2 (EBF2)*	40.7 ± 1.5	49.7 ± 1.2	41.6 ± 0.8	30.1 ± 0.0	50.8 ± 3.4	52.0 ± 5.0	0.3	-0.3	0.8	0	No impact	16%
**(C)Down-regulated ETC-specific DEGs**													
**Uniform wall specific**													
U2448	*VfEpsin 1 (EPN1)*	8.6 ± 0.9	3.8 ± 0.2	6.2 ± 0.7	3.6 ± 0.4	4.4 ± 0.7	3.8 ± 0.5	-1.2*	0.7	0.3	-0.2	24%	No impact
U8692	*Vfphosphatidylinositol4-phosphate 5-kinase 6 (PIP5K6)*	1.2 ± 0.3	0.4 ± 0.1	0.6 ± 0.2	0 ± 0	0 ± 0	0 ± 0	-1.6*	0.7	0	0	217%	No impact

Supplementary Table S6. Effect of vesicle trafficking on regulation of sterol distribution by $_{apo}H_2O_2$ signalling in *trans*-differentiating epidermal transfer cells (ETCs) of cultured *V. faba* cotyledons.

(A) Cotyledons were cultured on MS medium containing the $_{apo}H_2O_2$ scavenger, ascorbic acid (AA) for 9 h at 26°C, then washed in 3 x 5 min in dH_2O before being transferred to MS medium in the absence/presence of endocytosis inhibitor, Dynasore, for 4 h at 4°C and subsequently cultured for a further 6 h at 26°C.
(B) Cotyledons were cultured on MS medium alone for 9 h and then transferred to MS medium containing AA in the absence/presence of Dynasore for 4 h at 4°C and subsequently cultured for a further 6 h at 26°C. Thereafter, transverse sections of treated cotyledons were stained with Filipin. Fluorescence was measured as total pixels in specified regions of the ETC plasma membrane (PM). Data at each culture time are Means ± SEs from four replicate cotyledons; 20 cells per cotyledon.

Cotyledon treatment			Filipin fluorescence (arbitrary units) in ETC:			
			Outer periclinal PM	Anticlinal PM	Inner periclinal PM	Total
(A)	9 h AA (10 mM) +	6 h MS	133 ± 6	0 ± 0	6 ± 1	139 ± 6
		6 h MS + Dynasore (100 µM)	123 ± 8	0 ± 0	7 ± 2	130 ± 9
(B)	9 h MS +	6 h AA (10 mM)	46 ± 5	39 ± 3	45 ± 5	130 ± 8
		6 h AA (10 mM) + Dynasore (100 µM)	120 ± 4	6 ± 2	3 ± 1	129 ± 5

Supplementary Table S7. Impact of the $_{apo}H_2O_2$ scavenger, ascorbic acid (AA), on transcript abundance of (A) up-regulated epidermal transfer cell (ETC)-specific differentially expressed genes (DEGs) encoding proteins involved in vesicle trafficking, docking and exocytosis, (B) expressed genes (RPKM>1) encoding proteins involved in vesicle docking possibly regulated by $_{apo}H_2O_2$ at a post-translational level and (C) down-regulated ETC specific DEGs encoding proteins involved in endocytosis elevated by AA. ETC-specific DEGs met the following criteria: Number of uniquely mapped reads per kilo base per million reads (RPKM) of transcript in ETCs at 3 h or 12 h > 1; 2 fold change (Log2 FC) of > 1 with a false discovery rate (FDR) corrected P value < 0.05 calculated using LimmaR (Ritchie ME, Phipson B, Wu D, Hu Y, Law CW, Shi W, Smyth GK.2015. limma powers differential expression analyses for RNA-sequencing and microarray studies. Nucleic Acids Research 43, e47) between either 0 to 3 h (uniform wall layer specific DEGs), 3 to 12 h (wall ingrowth papillae specific DEGs) or sustained up-regulation from 0 to 3 h to 12 h of cotyledon culture (uniform wall layer/wall ingrowth papillae shared DEGs); DEGs detected in the ETCs were absent from the storage parenchyma cells (SPCs). In specified cases, where transcript abundance of genes was. significantly impacted by AA (P<0.05), these responses are presented as percentile changes compared to the control. Function of the encoded protein was inferred by the best-fit percentage amino acid alignment with the closest Arabidopsis homolog using TAIR10 and Araport 11 databases. RPKM values are means ± SE of 6 replicate batches of cotyledons in ETCs and 3 replicates in SPCs. DEGs indicated by asterisks.

Sequence ID	Name	Relative transcript level (RPKM) in:						Log_2 fold change in:				Sensitivity to AA at:	
		ETCs at:			SPC at:			ETCs at:		SPCs at:			
		0 h	3 h	12 h	0 h	3 h	12 h	0 to 3 h	3 to 12 h	0 to 3 h	3 to 12 h	3 h	12 h
**(A) Up-regulated ETC-specific DEGs**													
**Uniform wall layer specific**													
U9337	*Vfsyntaxin of plants32(SYP32)*	32.2 ± 2.0	73.6 ± 2.5	29.5 ± 1.0	15.3 ± 0.8	28.8 ± 2.0	14.2 ± 0.0	1.2*	-1.3*	0.9	-1.0	-51%	No impact
CL10226.C1	*Vfv-SNARE SEC22 (SEC22)*	50.4 ± 3.9	139.4 ± 8.9	60.8 ± 0.3	32.0 ± 1.6	63.2 ± 1.7	26.9 ± 1.0	1.5*	-1.2*	1.0	-1.2	-39%	No impact
CL1456.C1	*Vfβ-COP*	1.3 ± 0.1	4.2 ± 0.2	1.6 ± 0.1	1.1 ± 0.2	2.2 ± 0.4	0.9 ± 0.0	1.7*	-1.4*	1.0	-1.3	-41%	No impact
U9188	*Vfγ-COP*	27.6 ± 1.1	58.2 ± 4.5	22.4 ± 1.2	9.9 ± 1.8	17.2 ± 2.6	11.9 ± 1.1	1.1*	-1.4*	0.8	-0.5	-80%	No impact
CL3290.C2	*VfADP-ribosylation factorGTPase-activating domain protein5 (AGD5)*	1.1 ± 0.1	2.8 ± 0.1	1.0 ± 0.1	0.7 ± 0.0	1.4 ± 0.0	1.0 ± 0.1	1.3*	-1.5*	1.1	-0.5	-88%	No impact
U25327	*Vfsyntaxin of plants43(SYP43)*	3.6 ± 0.4	11.2 ± 0.2	3.3 ± 0.2	1.2 ± 0.1	2.6 ± 0.0	1.7 ± 0.2	1.6*	-1.8*	1.1	-0.6	-74%	No impact
U21554	*VfEXO70H7*	0.2 ± 0.0	1.3 ± 0.1	0.2 ± 0.0	0.1 ± 0.0	0.4 ± 0.0	0.2 ± 0.1	2.7*	-2.7*	1.8	-1.1	-77%	No impact
U18837	*Vfsyntaxin of plants121(SYP121)*	11.4 ± 1.8	47.1 ± 4.2	13.2 ±0.6	2.8 ± 0.2	7.6 ± 2.3	7.1 ± 2.3	2.0*	-1.8*	1.5	-0.1	-54%	No impact
**(B) Genes encoding vesicle docking proteins possibly post-translationally sensitive to $_{apo}H_2O_2$**													
U20773	*VfSynaptotagmin1 (SYT1)*	8.8 ± 0.7	11.0 ± 0.9	7.7 ± 0.8	2.4 ± 0.1	2.6 ± 0.0	3.6 ± 0.4	0.3	-0.5	0.1	0.5	No impact	No impact
U15557	*VfSynaptotagmin2 (SYT2)*	51.7 ± 2.7	42.4 ± 1.4	50.8 ± 1.4	30.0 ± 2.5	38.0 ± 2.0	26.7 ± 1.1	-0.3	0.3	0.3	-0.5	No impact	No impact
cl10577.C1	*VfSynaptotagmin3 (SYT3)*	1.6 ± 0.2	2.5 ± 0.2	3.5 ± 0.2	0.8 ± 0.1	1.5 ± 0.2	1.4 ± 0.3	0.6	0.5	0.8	-0.1	No impact	No impact
**(C) Down-regulated ETC specific DEGs**													
**Wall ingrowth papillae specific**													
U18257	*VfSH3P1 containing domain protein(SH3P1)*	15.9 ± 8.2	35.7 ± 12.2	2.4 ± 0.5	1.9 ± 0.8	27.7 ± 0.8	262.0 ± 45.4	1.2	-3.9*	3.9	3.2	No impact	490%
U8564	*Vfphosphatidylinositol4-phosphate 5-kinase 2 (PIP5K2)*	1.4 ± 0.3	1.0 ± 0.2	0.4 ± 0.1	1.3 ± 0.0	0.0 ± 0.0	0.2 ± 0.1	-0.5	-1.1*	-3.1*	0.0	No impact	127%
